# Disturbance Ecology Meets Bovine Tuberculosis (bTB) Epidemiology: A Before-and-After Study on the Association between Forest Clearfelling and bTB Herd Risk in Cattle Herds

**DOI:** 10.3390/pathogens11070807

**Published:** 2022-07-19

**Authors:** Andrew W. Byrne, Damien Barrett, Philip Breslin, James O’Keeffe, Kilian J. Murphy, Kimberly Conteddu, Virginia Morera-Pujol, Eoin Ryan, Simone Ciuti

**Affiliations:** 1 One Health Scientific Support Unit, Department of Agriculture, Food and the Marine, D02 WK12 Dublin, Ireland; damien.barrett@agriculture.gov.ie; 2 Ruminant Animal Health Division, Department of Agriculture, Food and the Marine, D02 WK12 Dublin, Ireland; philip.breslin@agriculture.gov.ie (P.B.); james.okeeffe@agriculture.gov.ie (J.O.); eoin.ryan@agriculture.gov.ie (E.R.); 3 Laboratory of Wildlife Ecology and Behaviour, School of Biology and Environmental Science, University College Dublin, D04 V1W8 Dublin, Ireland; kilian.murphy@ucdconnect.ie (K.J.M.); kimberly.conteddu@ucdconnect.ie (K.C.); virginia.morera-pujol@ucd.ie (V.M.-P.); simore.ciuti@ucd.ie (S.C.)

**Keywords:** wildlife disease, mycobacteria, zoonotic disease, anthropogenic disturbance, social perturbation

## Abstract

Disturbance ecology refers to the study of discrete processes that disrupt the structure or dynamics of an ecosystem. Such processes can, therefore, affect wildlife species ecology, including those that are important pathogen hosts. We report on an observational before-and-after study on the association between forest clearfelling and bovine tuberculosis (bTB) herd risk in cattle herds, an episystem where badgers (*Meles meles*) are the primary wildlife spillover host. The study design compared herd bTB breakdown risk for a period of 1 year prior to and after exposure to clearfelling across Ireland at sites cut in 2015–2017. The percent of herds positive rose from 3.47% prior to clearfelling to 4.08% after exposure. After controlling for confounders (e.g., herd size, herd type), we found that cattle herds significantly increased their odds of experiencing a bTB breakdown by 1.2-times (95%CIs: 1.07–1.36) up to 1 year after a clearfell risk period. Disturbance ecology of wildlife reservoirs is an understudied area with regards to shared endemic pathogens. Epidemiological observational studies are the first step in building an evidence base to assess the impact of such disturbance events; however, such studies are limited in inferring the mechanism for any changes in risk observed. The current cohort study suggested an association between clearfelling and bTB risk, which we speculate could relate to wildlife disturbance affecting pathogen spillback to cattle, though the study design precludes causal inference. Further studies are required. However, ultimately, integration of epidemiology with wildlife ecology will be important for understanding the underlying mechanisms involved, and to derive suitable effective management proposals, if required.

## 1. Introduction

The disturbance of ecosystems can lead to complex and non-linear effects to species, communities and ecological processes, including modulating disease dynamics [1,2,3,4]. Human-induced disturbance to wildlife populations, for example, through landscape modification, can result in changes in the prevalence of infectious diseases within wildlife hosts, impacting local wildlife population dynamics [5]. Increased habitat fragmentation can result in changing wildlife movement patterns, dispersal strategies [6] and stress [7], which could further increase the intraspecific spread of infection [5]. Furthermore, such disturbances can increase the probability of interactions between wildlife hosts, domestic hosts and, in the case of zoonotic pathogens, people, elevating exposure risk [2,8,9]. Direct exposure from wildlife to humans can occur through activities that directly expose people to wild hosts, such as deforestation or eating and processing bushmeat, for example, in the case of ebola [10]. Often, the spread of infections between wildlife populations and humans can be modulated by spillover events into domestic animal hosts [11,12]. Several examples of zoonotic pathogens shared between wildlife hosts and domestic hosts have been found to be impacted by ecosystem disturbances of different kinds and scales, for example, Hendra virus in fruit bats and horses [13,14].

Bovine tuberculosis is caused by *Mycobacterium bovis*, part of the *Mycobacterium tuberculosis* complex [15]. The pathogen remains endemic in several countries worldwide, including Ireland, despite long-term and intensive control programs [16,17]. Many risk analyses have been undertaken to assess what factors, from farm level to national level, are associated with bTB risk, maintenance and spread [16,18,19]. Common herd-level risk factors include herd size (and associated intensity-related metrics), herd history of infection, geography (spatial variation and localised clustering) and, importantly, the presence of a wildlife reservoir (e.g., the badger, *Meles meles*; [17,20]).

In Europe, the primary wildlife hosts of *M. bovis* include deer, wild boar and European badgers [21], though bovine tuberculosis can be a problem for other species of concern also, e.g., European bison in Poland [22]. In some areas, there is evidence of a multi-host community sharing infectious strains (e.g., parts of France, Spain, Poland; [21,23]). European badgers are the main wildlife reservoir of infection in Ireland and the United Kingdom. Evidence for interspecific transmission between badgers and cattle hosts has been demonstrated by whole genome sequencing data. These data provide evidence for the frequent transmission of infection between the two hosts (badgers and cattle) at very fine spatial and temporal scales [24,25]. Large-scale and replicated field studies have been conducted, where intensive repeated removal (culling) leads to measurable reductions in cattle herd bTB risk (e.g., [26,27]). In Britain, evidence suggests that culling activities are associated with disturbance in the socio-spatial arrangement of badger social groups, which has been hypothesized to be the mechanism underlying patterns of transient increasing risk of bTB in cattle herds surrounding cull zones [27].

### bTB in Ireland: A Perfect Storm of Opportunities to Understand the Link between Ecological Disturbance and Bovine Tuberculosis

The bTB-wildlife-cattle episystem (i.e., the whole complement of biological and environmental components of the disease system [28]) in Ireland is an excellent study case to explore the dynamics of wildlife disease maintenance, spread and spillover to domestic hosts and how it is modulated by landscape disturbance [29], due to the extensive data available on the disease and the attempts to control infection in both cattle and wildlife [30].

In Ireland, bTB remains endemic and a priority pathogen of cattle herds, despite a costly multi-decadal control campaign [31]. Badgers are widespread and abundant [17,32], occupying a niche space that brings them into contact directly (though infrequently) and indirectly with cattle [33]. Where bTB is endemic in badger populations, infection is widely geographically disseminated and local hotspots of infection can exceed 30% prevalence [34,35]. Badgers are considered a widespread risk to cattle herds in the endemic area, and consequently, in Ireland, there has been a national badger culling policy in response to this since 2004, and more recently, a BCG vaccination program [30]. However, deer have also been speculated to be another wildlife risk to cattle herds, especially in local areas where deer densities are high and/or increasing [17,31,36].

Forest cover accounts for approximately 11% of the Irish landscape, with afforestation policies leading to expansive growth in recent decades [37]. The majority (~74%) of the forest stock being <30 years old [37] with publicly managed stock accounts for approximately 51% of total forest cover [38]. With this expansion of forest, there is greater probability of pasture-dominated landscapes that share perimeters with forest stands now than in the past. Forest cover is an important habitat type for badgers, especially near perimeters of larger forest stands near pasture (as rich pasture can be a source of invertebrate foraging; [39]). In addition, the relative amount of forest cover directly correlates with deer distribution and relative abundance in Ireland [40,41]. While data are currently sparse, there is evidence to suggest that deer species have significantly increased their spatial distributions [40] and potentially abundance [41,42] in recent decades in Ireland.

Clearfelling is the removal of forest stands of all marketable trees at the end of a forest rotation (usually after ~30–50 years, depending on the species) and has been associated with disturbance of population dynamics and movement ecology of resident mammal species in some countries [43,44,45]. However, ecological interventions are put in place to reduce this risk, for example, mapping badger burrows (setts) and refraining from harvesting within a determined perimeter [38]. The disturbance of reservoir populations can lead to spillover infection from wild animals to domestic hosts [46] and this may be through mechanisms involving increasing cattle–wildlife interactions (contact patterns), spatial ranging patterns of infected wildlife hosts (indirect spread) or stress-induced increase in transmission (pathological progression).

Increasing evidence suggests that the interface between habitats can be an important contact point for the spillover of pathogens between wildlife and domestic hosts [47]. Therefore, we hypothesized that disturbance to forest coverage via clearfelling activity on wildlife hosts may be associated with an increased risk of bTB in herds in close vicinity to such activities. Our correlative approach does not shed light on the mechanism for such changes in risk, but instead is seen as a first step to evaluate the hypothesis and an indicator as to whether further investigations are justified in a resource-limited research agenda.

## 2. Results

### 2.1. Sample Sizes and Breakdown Descriptive Statistics

**Figure 1:**
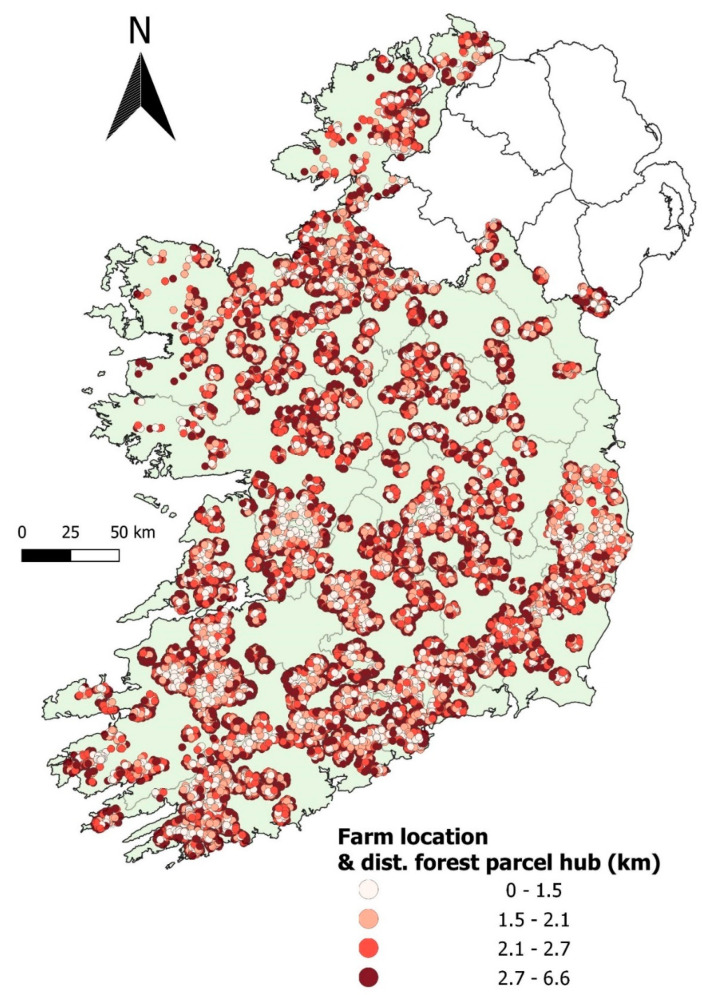
Locations of the herds and their distance to the centroid of their associated clearfell site.

The final dataset had 16,380 herds that met our inclusion criteria, which are mapped in Figure 1. As the study design was a before-and-after study, our full dataset included 32,760 observations (i.e., a pre-clearfell record and a post-clearfell record for each herd). Table 1 presents the 2 × 2 table for the proportion of herds that disclosed one or more reactors during the pre-clearfell and post-clearfell exposure periods. During the pre-clearfell period, 3.47% (exact binomial 95%CI: 3.20–3.77%) of herds disclosed with one or more reactors; post clearfell, this proportion increased to 4.08% (exact 95%CI: 3.79–4.40%). A McNemar’s χ^2^ test suggested there was an association between TB status and pre/post-clearfell period (Exact McNemar χ^2^; Pr = 0.002).

When invoking a threshold of >2 reactors to define a ‘breakdown’, the pre-clearfell prevalence was 1.55% (exact 95%CI: 1.37–1.75) and the post-clearfell prevalence was 1.81% (exact 95%CI: 1.61–2.02%); this subtle difference was ‘non-significant’ using a univariable unadjusted McNemar’s χ^2^ test (Exact McNemar χ^2^; Pr = 0.057).

### 2.2. Multivariable Binomial Model

The final multi-level mixed effects logit regression model is presented in Table 2, where herd and county are controlled as random effects. In total, there were 24 county groupings, with an average of 1365 observations (range: 376–5022), within which 16,380 herds were modelled. Overall, the model explained a significant amount of variation relative to a model without independent predictors (log likelihood = −4919.87; prob > χ^2^ < 0.001), and there was evidence of clustering effects at the county and herd levels (LR test vs. logistic model: χ^2^ (DF:2) = 258.64; prob > χ^2^ = 0.0001). The final model included the primary predictor of interest (dummy variable representing pre- or post-clearance), log-herd size, herd type and the proportion of the landscape (radius 3 km) of herd that had managed forest. There was no evidence to suggest that other variables (e.g., distance to clearfell, perimeter of forest, perimeter of clearfell) were associated with bTB herd risk, with *p*-values being greater than the alpha (*p* > 0.05), the 95%CI of the parameter estimates straddling 1 (for odds ratio) and models not improving with the inclusion of variables (using AIC as a metric).

bTB risk increased with increasing log-herd size (OR: 1.76; 95%CI: 1.62–1.90), increasing proportion of local area forested (OR: 2.28; 95%CI: 1.07–4.85), and if the herd-type designation was dairy relative to beef (OR: 2.28; 95%CI: 1.12–1.76). There was no evidence of suckler herds (farms where suckler cows are kept for beef production, with calves kept with dams in herds until being sold for fattening) differing in risk relative to beef herds (OR: 1.00; 95%CI: 0.83–1.21); however, ‘other’ herds appeared to exhibit lower risk than beef herds (OR: 0.48; 95%CI: 0.33–0.70).

The odds of a herd experiencing a bTB breakdown were 1.20 (95%CI: 1.07–1.36) post a clearfell event, relative to the same cohort of herds during the pre-clearfell exposure period.

As this model was developed in a generalised linear model framework, the parameters are subject-specific parameters (i.e., the parameter refers to the odds of a herd being TB-positive post-clearfell, relative to the same herd pre-clearfell). To explore whether there was any meaningful difference with a population-averaged model (i.e., the parameter then refers to the odds of an average herd being TB-positive post-clearfell, relative to the average herd risk post-clearfell), we fitted a Generalised Estimating Equations (GEE), with clustering on herd_id, and county fitted as a fixed effect. Overall, the parameter estimates were very similar to the mixed linear model (compare Table 2 with Table S1) and had no effect on the interpretation of the model inferences.

To assess the stability of positive association between bTB risk post clearfelling as a post-hoc test, a model with an interaction term between pre-post clearance and county was fitted. This model allowed us to assess whether the direction of the effect was the same across counties or whether the pattern was driven by certain counties. The results suggested that there was a positive association for all counties, with the exception of Carlow, Kilkenny and Roscommon (Figure S2). However, despite negative point parameter estimates, the effect of forest clearance for each of these counties was non-significantly different from zero (interaction terms were: Carlow OR: 0.60; *p* = 0.253; 95%CI: 0.25–1.44; Kilkenny OR: 0.66; *p* = 0.447; 95%CI: 0.23–1.91; Roscommon OR: 0.65; *p* = 0.479; 95%CI: 0.20–2.14).

Finally, when we fitted a model with breakdowns of >2 reactors, the impact of the clearfell event was positive but non-significant (*p* = 0.061). The 95%ci of the odds ratio for the mixed effects model straddled 1 (0.992–1.419; see Supplementary Material for details). Similar results were found when the model was fitted using a GEE model (Supplementary Material).

### 2.3. Temporal Trend Analysis

**Figure 2:**
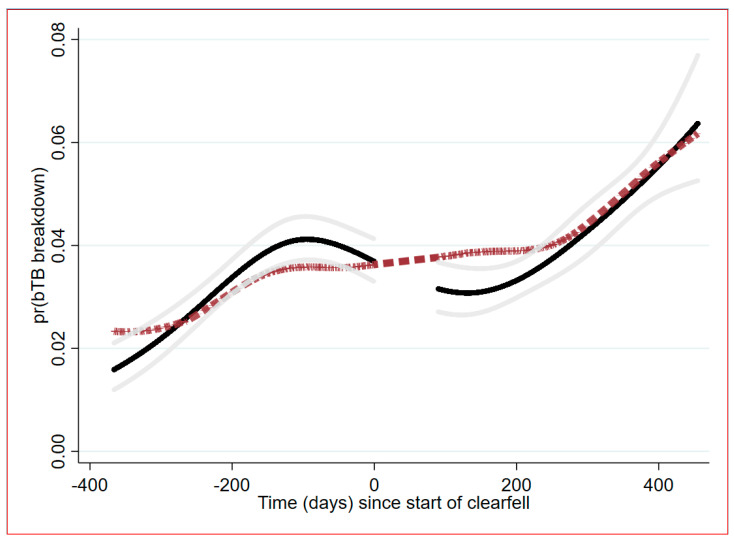
Time series plot of the estimated probability of bTB breakdown failure for cattle herd prior to (before day 0) and after a forest clearfell (after day 90). Dashed line output from a locally weighted regression (LOWESS); black line is the mean predicted probability of failure from a cubic spline model with associated 95%CI (grey lines).

**Figure 3:**
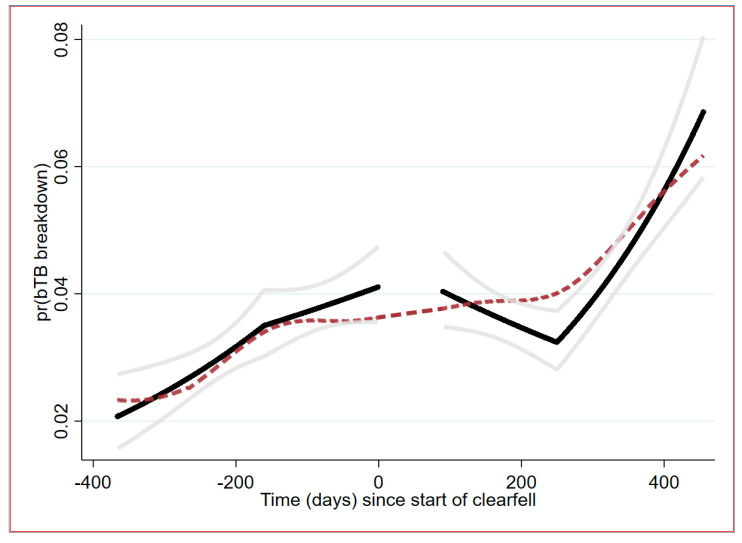
Time series plot of the estimated probability of bTB breakdown failure for cattle herd prior to (before day 0) and after a forest clearfell (after day 90). Dashed line output from a locally weighted regression (LOWESS); black line is the mean predicted probability of failure from a linear spline model with associated 95%CI (grey lines).

The locally weighted regression (LOWESS) algorithm suggested that the temporal trend across the interrupted time series was composed of a gradual mean increase in risk up to around 200 days pre-clearfell commencement. There was a plateauing effect between day −200 and day 0, and again from day 90 through to day 200, when there was an increasing temporal trend in the breakdown risk of exposed herds. Two different spline models fitted (linear and cubic splines) to the time series also suggested periods of increasing risk early in the study period (<−200 days) and later in the study period (<200 days; Figure 2 and Figure 3). Knots, based on quartiles of observations across relative time, split the time series into four segments on days −366 to −161, −161 to 0, 90 to 250, 250 to 455. The linear spline model suggested a significant increasing trend in spline 1 (i.e., the slope of the trend was significantly different to 0; OR: 1.003 per day; *p* = 0.004), a non-signifcant increasing trend in spline 2 (OR: 1.001; *p* = 0.168), a significant decreasing trend in spline 3 (OR: 0.999; *p* = 0.047), and a final significant increasing trend for spline 4 (OR: 1.004; *p* =< 0.001). Estimating the same model with piecewise (marginal) parameter comparisons suggested that the slope of spline 2 did not significantly change relative to spline 1 (OR: 0.998; *p* = 0.283) and that the slope of spline 3 did not significantly differ from spline 2 (OR: 0.998; *p* = 0.077). However, there was a significant increase between spline 3 and spline 4 (OR: 1.005; *p* < 0.001).

Using estimates from the cubic spline model, the odds of breakdown were 3.294 (95%CI: 2.425–4.473) higher for the average herd one-year after clearfelling (day 365) relative to herd risk one-year prior to clear-felling (day −365; *p* < 0.001). The odds ratio diminished to 1.393 (95%CI: 1.184–1.641; *p* < 0.001), comparing the average herd at one year after clearfelling, relative to the average herd the day before clear felling commencement (day −1).

## 3. Discussion

Recent research suggested that anthropogenic disturbance of ecosystems can have effects on spillover infection from wild populations to domestic animal hosts and/or human populations, in a range of episystems [1,2,4]. Our preliminary analysis provides some support to the hypothesis that there was an association between forest clearfelling and temporal risk profiles for bovine tuberculosis in adjoining cattle herds. Our data suggested that there was a significant increase in risk of breakdowns with one or more reactors during the post-clearfell period, relative to the pre-clearfell period. However, the increasing risk post-clearfell when using a threshold of more than two reactors was not significant. Our models also suggested that bTB risk was increased in areas with higher amounts of forested land. The temporal trends in the interrupted time series before and after the clearfell suggested that there was an increasing risk to herds both before and after the event. However, the rate of change was higher after the clearfell event, relative to before the clearfell, especially after day 250 post-clearfell commencement.

The mechanism for these patterns cannot be inferred from these retrospective models, but we speculate that it may involve the disturbance of wildlife [48]. Badgers (*Meles meles*) and, to a lesser extent, deer species, are a known wildlife host of bTB and are involved in the epidemiology of infection in local cattle herds [26,32,36,49]. Disturbances, such as road building and badger culling, are known to affect the socio-spatial structure of badger populations and can lead to the increased movement of animals amongst social groups (e.g., [50,51,52]). In Britain, culling disturbances have been associated with temporary increased risk of bTB spread to cattle herds in adjoining lands (e.g., [27,53]). Recently, Barroso et al. [48] provided spatio-temporal evidence of an associated risk of bTB breakdown for herds in close proximity to a newly built motorway in Ireland. However, the mechanistic underpinning of this study, and our present study, cannot be inferred given the available data. Importantly, data on the effects of disturbances, such as clearfelling and road building, need to be established, as well as how this relates to interspecific transmission. Gaughran et al. [52] and O’Hagan et al. [54] both used GPS trackers on badgers to assess the impact of road realignment and low-level culling on ranging behaviour, respectively. Both studies showed that such processes can have subtle effects on badger behaviour, which may not meaningfully contribute to increasing disease spread dynamics. Other approaches to measuring changes in population dynamics, including mark–recapture and genetic relatedness, have also recently failed to show large population-level changes because of small-scale localized badger culling [55]. However, a process like clear-felling could arguably have a greater impact on local badger dynamics due to the acute impact on the local landscape—with the caveat of risk mitigation measures being put in place, such as leaving non-cleared areas around badger setts in clearfelled sites. The actual threshold before disturbance may manifest into a measurable change in infectious disease transmission is an open question.

Wright et al. [56] found a correlation between metrics of badger sett (burrow) disturbance and area-level cattle herd TB risk. “Disturbance” in that study included signs of persecution (e.g., digging at setts, pumping setts with slurry) and building construction activities. Areas projected to have higher levels of persecution, using sampled sites and kriging to interpolate between sites, were associated with higher levels of bTB in cattle herds [56]. However, the direction of association, that is, whether disturbance increased bTB risk via animal perturbations or higher bTB risk experienced by farmers caused increased wildlife reservoir disturbance via illegal persecution, was not clearly established.

We found that the area of managed forest in the vicinity of the farmstead was correlated with increasing herd bTB breakdown risk. Speculatively, this could suggest that herds that are at greater exposure to such habitats may be associated with increased bTB risk, presumably from wildlife spillover effects [4]. Indeed, the interface between habitats can be a point of spillover of infection to domestic animal hosts [4,57].

We found significant associations between herd risk and herd size, which is very common for bTB models [17,18]. Herd size can be a proxy for agricultural intensity and will often correlate with the number of farm fragments and geographical area [58,59]. Such large herds can also have exposure from many neighbours [59], increasing the probability for neighbourhood spread. Dairy herds were found to be of higher bTB risk, relative to beef production herds. Dairy herds tend to be larger in Ireland than beef herds and have an older age profile. Age can be an individual-level risk factor for bTB, given the animals cumulative time at risk. Dairy production can also be phyiologically intense on cows, which could increase susceptibility if exposed. There could also be genetic elements, related to dairy breeds, that could affect bTB susceptibility and infectivity [60].

This was the first observational approach to assess the relationship between clearfelling and bTB risk, and we acknowledge the limitations and efforts made to correct for some methodological limitations. Firstly, the before-and-after study design limits inference regarding causation. However, we included a temporal trend analysis to help improve upon the robustness of the analytical approach [61,62]. The national trends indicated that there was an overall increase in both incidence and prevalence from 2014 to 2019 (Supplementary Figures S3 and S4), even though the animal-level test failures per 1000 test slightly decreased over the period (Figure S4). This suggests caution should be applied to interpreting our outcome; however, national figures mask considerable county-level variation in incidence over time (Figure S4). The use of homestead spatial location to identify exposed farms is also limiting in that the fragmented nature of farms in Ireland means there may have been some herds with low exposure. This would have pushed our model outcome towards to the null, therefore, decreasing its ability to find a true effect. The study would have benefited from contemporaneous wildlife covariates and will be used in future planned studies. Previous research using spatial models of badger abundance in Ireland for cattle herd risk estimation showed how badger culling effects can modulate breakdown risk [32]. The variation in badger abundance is further complicated with the advent of widespread vaccination instead of culling [30], which will require additional autecological studies of badger populations to predict variations in abundance, population growth and movement dynamics. Finally, there are limited data on deer available in Ireland; however, improved wildlife abundance estimates are due to be published shortly (S. Ciuti, pers. Com.), providing greater ability to understand the community dynamics at play impacting on infectious disease spillover.

Given these initial findings, and the limitations with the current study’s approach, a matched case-control study of Irish farms exposed to forest clearance was developed [29]. The study design was designed to gain insights into how local ecological conditions modulated bTB risk at various spatio-temporal scales after a clearfell event. That study found an association between clearfelling forestry and cattle herd bTB risk; however, the effects could be either positive or negative, dependent on many factors, e.g., the size of the clearfell (in hectares), the distance to the farm, the time elapsed since the clearfell occurred and, perhaps most importantly, the area (in hectares) of natural habitats around the farm, which may act as refugia for wildlife vectors. The changing dynamics of bTB risk over space and time shown by Murphy et al. [29] and the findings of the present study clearly demonstrate the complexity of elucidating disease risk in response to ecological disturbance and highlight the need for these challenges to be approached from multiple different perspectives (e.g., ecological, epidemiological, veterinary science), with different study designs, to build a complete understanding of these processes in order to inform policy and management of the agro-ecological episystem in Ireland.

Future studies relating to the wildlife aspect of bTB spillover are advocated to gain greater understanding of the link between disturbance and any resulting effects on bTB risk for farms in their vicinity. Studies focusing on informing wildlife management and policy could be beneficial to stakeholders for risk mitigation. This disturbance ecology paradigm moving forward would benefit from the use of wildlife-tracking technology (e.g., GPS collars, accelerometers) and paired with other devices (e.g., camera traps and acoustic recorders) to estimate wildlife state variables in response to disturbance [63]. Studies that integrate disparate data sources could help parameterise social network analyses to inform on inter/intra-species interactions after disturbances [64] and move to more mechanistic explanations of the results found during observational studies. Finally, simulation studies should be used to test the efficacy of management and policy and for informing future field data collection [65,66].

## 4. Conclusions

The present study found mixed evidence of an association between bTB breakdown risk and the timing of clearfelling activities in Ireland, for herds with farmsteads within 3 km. The association was significant for a model, defining breakdowns as those with one or more reactors disclosed. However, the association was non-significant for a model where only breakdowns with two or more reactors were included as positive cases. A time series assessment using spline models suggested that there was a significant increase in risk across the time period; however, the rate of change (slope) increased significantly after day 250 post-clearfelling initiation. These early data support additional investment in further studies to better establish causation and build up a better mechanistic understanding of this disturbance spillover system.

## 5. Methods

### 5.1. Study Design

The study design was a retrospective, observational, single-arm, before-and-after (as called pre-post study) intervention study [67]. Such study designs are appropriate when rapidly trying to establish whether there is an association between an “intervention” by comparing an exposed group prior to and after the event, where the exposed groups become their own control comparisons. The event in this case was an unplanned event, a clear-felling operation, that we hypothesised to be associated with our outcome. In this study, we assessed the potential effect of clearfelling on local TB risk in cattle herds by (i) developing a model to test the hypothesis that mean TB risk, while accounting for repeat measures, increases during a post-event risk period relative to a pre-event risk period; and by (ii) fitting time-series regression models to the pre-event and post-event risk windows. Applying additional time series analytic techniques can overcome some of the problems relating to temporal trends in the data [61], as has been applied recently to a veterinary example [62].

### 5.2. Definitions

The outcome of interest was whether a herd experienced a bovine TB breakdown. A bTB breakdown is the disclosure of “reactors” to the comparative tuberculin skin test. In Ireland, all herds are subjected to an annual test. This means all animals (>42 days old) residing in a particular herd will receive at least one antemortem test from the annual round test. Test failure (i.e., reactor disclosure) results in the removal and slaughter of test-positive cattle. For the purposes of this study we developed two models for bTB breakdown, defined as a herd with ≥1 or >2 standard reactors. The purpose of analysing the data in two ways was due to the imperfect nature of the skin test [68]. The latter approach has been employed previously (e.g., [48]) to avoid the scenario where some single reactor breakdowns result from false-positives due to imperfect herd-level specificity of the skin test. All data on herd testing were gathered from a national database, the Animal Health Computer System (AHCS).

The primary predictor of interest was whether the time at risk was prior to or after a clearfell event. A clearfell event was the harvesting of one or more forest stands (Harvest Units) from a managed forest from a state forest management company (Coillte; https://www.coillte.ie/; accessed on 18 July 2022). Clearfell is the most common silvicultural system practiced in plantations in Ireland and the UK and involves the removal of all marketable trees from an area at the end of the rotation (usually at between 30 to 50 years of age). Approximately 7442 ha were clearfelled in Ireland in 2019 (DAFM 2020). Prior to clearfell, forest stands are managed through the forest cycle/rotation, which includes tending, thinning and engineering management activities. It should be noted that all forest stands are surveyed for wildlife prior to clearfelling and mitigation strategies undertaken (DAFM 2019). National standards outline that as part of each Harvest Plan important wildlife habitats are identified, marked, mapped and retained with an effective exclusion zone (e.g., 20 m exclusion zone around setts that is undisturbed during clearfelling), and in consultation with appropriate statutory bodies, such as the National Parks and Wildlife Service (NPWS).

Based on the movement ecology of badgers in Ireland, herds within 3 km of a clearfell (via the location of farmstead) were recruited into the study [52,69,70]. Furthermore, 3 km or under is also the reported spatial scale of “perturbation” effects of badger culling (e.g., [27,53]).

### 5.3. Time at Risk

The risk of herd breakdown was assessed for one year (365 days) prior to and a one-year period post the clearfell event. Clearfell events occurred between 2015 and 2017 (inclusive). As there are several biological steps to be undertaken for the hypothesized mechanism for increasing risk due to clearfelling to occur, a post-clearfell period did not start for 3 months (90 days) after clearfell initiation. Therefore, the intervening period was not included in this analysis in an attempt to avoid issues raised during pervious wildlife TB intervention research regarding “biological plausibility” (see discussion in More et al. [71]). More et al. [71] highlighted that in order to measure a “perturbation” impact on wildlife in cattle herds’ bTB status, the following chain of events needs to occur: disruption in badger social organisation, dispersal of badgers infected with *Mycobacterium bovis*, contact and transmission between cattle and badgers and detection of infection within the cattle herd. Another reason for the lag is due to clearfelling activities not being instantaneous processes. Instead, it may take a period of time to complete the harvest while undertaking all the steps required to comply with national standards for felling [38,72].

Herds were included if:

- A whole herd test was completed between −366 days and −1 day prior to the first day of the month during which clearfelling occurred (note, clearfelling activities were recorded to the month/year level only);

and

**Figure 4:**
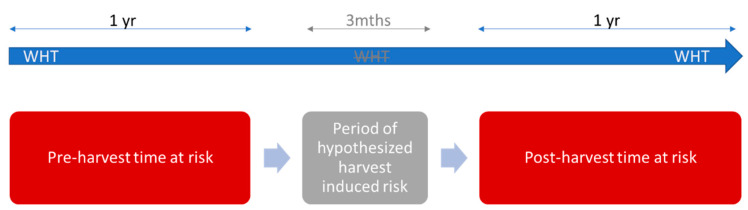
Schematic diagram of the time at risk measured during the pre-post study assessing whether there was any relationship between bTB herd risk and forest clear-felling. bTB surveillance during the risk period was assessed via whole-herd test (WHT).

- a whole herd test was completed between 90 days and 455 days after the first day of the month during which clearfelling occurred (i.e., 1 day to 365 days after the 3-month period of hypothesized harvest induced risk; Figure 4

### 5.4. Confounders

To enable the development of parsimonious models, a minimum set of confounders were employed in this study. The minimum confounder set for bTB in a number of herd-level risk factor studies includes herd size, herd type and herd TB history (e.g., [26,73]).

The herd size was estimated from whole-herd test records for the period of risk exposure, with each herd attributed the mean herd size over the study period. Herd type was assigned a metric recorded in the AHCS system, assigning each herd with a ‘beef’, ‘dairy’, ‘suckler’ and ‘other’ designation.

In addition to herd confounders, there is also the risk of differential exposure to wildlife from forest cover in the surrounding landscape. Furthermore, we hypothesized that the risk of TB from clearfelling disturbance could be ameliorated if there were a lot of ‘sink’ habitats into which wildlife may disperse. Alternatively, a lack of surrounding forest may result in increased risk of ‘perturbation’ effects, as animals may need to migrate further distances to find cover. Due to this, we added a variable which measured the proportion of surrounding areas of herds with forest coverage, using all non-felled forest stands within a 3 km radius of a herd (proportion forest). This variable was constructed using the QGIS environment. Buffers from the farmstead were constructed that measure the overlap between the forestry polygon and these buffers were generated as proportions.

In addition, edge effects may have a relationship with exposure risk (see discussion in [47]). Therefore, we also fitted models with the perimeter of forest and the perimeter of clearfell calculated as the perimeter of the geometry of the forest and clearfell polygons, respectively, that overlapped the 3 km buffers from each respective farmstead. Finally, though we made 3 km the a priori distance inclusion threshold, we also fitted a metric of distance from farmstead to clearfelled area, by including the Euclidean distance from the farmstead to the clearfell parcel centroid.

### 5.5. Descriptive Analysis

Comparisons were made between the proportion of herds that experienced a bTB breakdown during a whole-herd test during the risk windows pre- and post-clearfell. A 2 × 2 table was generated and the univariable exact test of proportions compared using a McNemar’s χ^2^ test, given the paired/repeated measures nature of the data.

### 5.6. Modelling bTB Risk: Binomial Regressions

We fitted regression models with binomial distribution of errors and logit link function. The basic model had the following form:

logit(0/1) ~ intercept + β_1_∗(before/after clearfell dummy variable) + β_2_ x2 + … + β_n_ xn + random effect_(herd_number)_ + error,

where β _1–n_ are the coefficients associated to predictors estimated by the model.

In this model, the outcome included all the binary statuses of all observations both before and after the clear-felling activity. Therefore, the model was testing the hypothesis that the proportion of herds breaking down in the vicinity of clearfelling was higher after the stand was cut than prior to the stand being cut. A dummy variable representing whether each observation was a ‘prior’ or ‘post’ harvest was our independent variable of interest. As the post-harvest observations were not totally independent of the prior-harvest observations, a random effect for herd was included.

It is possible that some variation in risk could be related to spatial variation and, therefore, ‘county’ was included in the model. County was fitted in two ways, one as a random effect, fitted as a multi-level model, with herds being nested within counties (herd_id < county), using the XTMELOGIT routine in Stata 15. In addition, population-averaged Generalised Estimating Equation (GEE) models were developed for comparative purposes, where county was fitted as a fixed effect. Model building was achieved using backwards elimination, using Akaike’s Information Criterion (AIC) to compare competing models [74]. Models with lower AIC were considered preferred models.

After model building, we applied a post-hoc assessment of whether the direction of association clearfelling and breakdown risk was consistent across all counties. To do this, an interaction term between the pre-post dummy variable and county was fitted. Parameter estimates and predictions from this model were used to assess whether there were county-level deviations from the population-level results.

### 5.7. Modelling bTB Risk: Time-Series Regressions

The second modelling approach was to assess the time-series during the two risk periods prior and post the clearfelling intervention. To visualise the data, we first used a local regression technique, Locally Weighted Scatterplot Smoothing (LOWESS), the dependent variable being the binary herd-level test result, with the single independent variable being the date of the herd test. We then fitted linear and restricted cubic splines to the time-series data. Default cut-points at each of the quartiles of the observations were determined using the MKSPLINE suite of tools in Stata 15.

## Figures and Tables

**Table 1:** Tabulation of the bTB statuses of 16,380 cattle herds within 3 km of pre- and post-clearfelling of forest stand during 2015–2017 in Ireland. Herd breakdown status was defined as either 1 or more reactors or >1 reactors, respectively.

	*≥1 Reactor Threshold*		*>2 Reactor Threshold*	
*Breakdown status*	Pre-	Post-	Pre-	Post-
*0*	15,811	15,711	16,126	16,084
*%*	96.53	95.92	98.45	98.19
*1*	569	669	254	296
*%*	3.47	4.08	1.55	1.81
*Total*	16,380	16,380	16,380	16,380

**Table 2:** Random effects logit model for the risk of disclosing 1 or more reactors post-forest clearfelling relative to pre-clearfelling in Ireland 2015–2018.

Parameter	OR	SE	Z	*p*	Upper 95%CI	Lower 95%CI
**Pre/Post**	1.204	0.074	3.020	0.003	1.067	1.357
**Log(Herd Size)**	1.758	0.072	13.750	0.000	1.622	1.905
**Beef (Ref)**						
**Dairy**	1.403	0.163	2.920	0.004	1.118	1.761
**Other**	0.483	0.092	−3.830	0.000	0.332	0.701
**Suckler**	1.002	0.097	0.020	0.981	0.829	1.212
**Proportion Forestry**	2.282	0.878	2.140	0.032	1.074	4.851
**Constant**	0.002	0.000	−28.880	0.000	0.001	0.003

## Data Availability

Data available upon request due to restrictions, e.g., privacy or ethical. The data presented in this study are available upon reasonable request from the corresponding authors, subject to legislative restrictions.

## Supplementary Materials

The following supporting information can be downloaded at: https://www.mdpi.com/article/10.3390/pathogens11070807/s1, Table S1. Population averaged Generalised Estimating Equations model of the risk of disclosing 1 or more reactors post forest clearfelling relative to pre-clearfelling in Ireland 2015–2018; Table S2: Mixed effect model with >2 reactors as breakdown threshold; Table S3: GEE model with >2 reactor threshold; Figure S1: Variation in estimated risk of one or more bTB reactor being disclosed within cattle herds across counties prior-to and post a clearfelling forestry event in the vicinity (3km search radius); Figure S2: Relationship between bTB risk pre and post forest clearance activities estimated demonstrated 12 counties in Ireland. Three counties (Carlow = CW, Kilkenny = KK, and Roscommon = RN) had significantly different trend to all other counties modelled (total n = 24); Figure S3: Time series plot of the estimated probability of bTB breakdown failure for cattle herd prior to (before day 0) and after a forest clearfell (after day 90). Dashed line (green) output from a locally weighted regression (LOWESS); red line is the mean predicted probability of failure from a cubic spline model; black line is the mean predicted probability of failure from a linear spline model; Figure S4: National trend in bTB herd incidence from year 2014–2019. The red dashed line is the overall country trend. Counties included were the most represented within the dataset with >3000 records each; Figure S5: National trend in bTB herd prevalence (orange) and individual level test failures per 1000 tests (blue) from year 2014–2019; Table S4: Example code and sample data (n = 1000 herds; 2000 observations). Base univariable model: xi: xtmelogit outcome_status i.pre_post ||herd_no:, or intpoints(12) level(95), where outcome_status is whether the herd experienced a breakdown during the observation period; pre_post is a binary variable representing whether the observation period was prior to or after a clearfell event; herd_no is an anonymised marker identifier variable for each herd.

## References

[1] Blitzer E.J., Dormann C.F., Holzschuh A., Klein A.M., Rand T.A., Tscharntke T. (2012). Spillover of functionally important organisms between managed and natural habitats. Agric. Ecosyst. Environ..

[2] Plowright R.K., Reaser J.K., Locke H., Woodley S.J., Patz J.A., Becker D.J., Oppler G., Hudson P.J., Tabor G.M. (2021). Land use-induced spillover: A call to action to safeguard environmental, animal, and human health. Lancet Planet. Health.

[3] Tompkins D.M., Dunn A., Smith M.J., Telfer S. (2011). Wildlife diseases: From individuals to ecosystems. J. Anim. Ecol..

[4] Faust C.L., McCallum H.I., Bloomfield L.S.P., Gottdenker N.L., Gillespie T.R., Torney C.J., Dobson A.P., Plowright R.K. (2018). Pathogen spillover during land conversion. Ecol. Lett..

[5] Brearley G., Rhodes J., Bradley A., Baxter G., Seabrook L., Lunney D., Liu Y., McAlpine C. (2013). Wildlife disease prevalence in human-modified landscapes. Biol. Rev..

[6] McCallum H., Dobson A. (2002). Disease, habitat fragmentation and conservation. Proc. R. Soc. Lond. Ser. B Biol. Sci..

[7] Hing S., Narayan E.J., Thompson R.C.A., Godfrey S.S. (2016). The relationship between physiological stress and wildlife disease: Consequences for health and conservation. Wildl. Res..

[8] Gibb R., Redding D.W., Chin K.Q., Donnelly C.A., Blackburn T.M., Newbold T., Jones K.E. (2020). Zoonotic host diversity increases in human-dominated ecosystems. Nature.

[9] Glidden C.K., Nova N., Kain M.P., Lagerstrom K.M., Skinner E.B., Mandle L., Sokolow S.H., Plowright R.K., Dirzo R., De Leo G.A. (2021). Human-mediated impacts on biodiversity and the consequences for zoonotic disease spillover. Curr. Biol..

[10] Alexander K.A., Sanderson C.E., Marathe M., Lewis B.L., Rivers C.M., Shaman J., Drake J.M., Lofgren E., Dato V.M., Eisenberg M.C. (2015). What factors might have led to the emergence of Ebola in West Africa?. PLoS Negl. Trop. Dis..

[11] Morens D.M., Fauci A.S. (2013). Emerging Infectious Diseases: Threats to Human Health and Global Stability. PLOS Pathog..

[12] Johnson C.K., Hitchens P., Evans T.S., Goldstein T., Thomas K., Clements A., Joly D.O., Wolfe N.D., Daszak P., Karesh W. (2015). Spillover and pandemic properties of zoonotic viruses with high host plasticity. Sci. Rep..

[13] Plowright R.K., Foley P., Field H.E., Dobson A.P., Foley J.E., Eby P., Daszak P. (2011). Urban habituation, ecological connectivity and epidemic dampening: The emergence of Hendra virus from flying foxes (*Pteropus* spp.). Proc. R. Soc. B Biol. Sci..

[14] Edson D., Field H., McMichael L., Jordan D., Kung N., Mayer D., Smith C. (2015). Flying-Fox Roost Disturbance and Hendra Virus Spillover Risk. PLoS ONE.

[15] Riojas M.A., McGough K.J., Rider-Riojas C.J., Rastogi N., Hazbón M.H. (2018). Phylogenomic analysis of the species of the Mycobacterium tuberculosis complex demonstrates that Mycobacterium africanum, Mycobacterium bovis, Mycobacterium caprae, Mycobacterium microti and Mycobacterium pinnipedii are later heterotypic synonyms of Mycobacterium tuberculosis. Int. J. Syst. Evol..

[16] Humblet M.F., Boschiroli M.L., Saegerman C. (2009). Classification of worldwide bovine tuberculosis risk factors in cattle: A stratified approach. Vet. Res..

[17] Allen A.R., Skuce R.A., Byrne A. (2018). Bovine Tuberculosis in Britain and Ireland—A Perfect Storm? the Confluence of Potential Ecological and Epidemiological Impediments to Controlling a Chronic Infectious Disease. Front. Vet. Sci..

[18] Skuce R.A., Allen A.R., McDowell S. (2012). Herd-Level Risk Factors for Bovine Tuberculosis: A Literature Review. Vet. Med. Int..

[19] Broughan J.M., Judge J., Ely E., Delahay R.J., Wilson G., Clifton-Hadley R.S., Goodchild A.V., Bishop H., Parry J.E., Downs S.H. (2016). A review of risk factors for bovine tuberculosis infection in cattle in the UK and Ireland. Epidemiol. Infect..

[20] Fitzgerald S.D., Kaneene J.B. (2013). Wildlife reservoirs of bovine tuberculosis worldwide: Hosts, pathology, surveillance, and control. Vet. Path..

[21] Gortázar C., Delahay R.J., Mcdonald R.A., Boadella M., Wilson G.J., Gavier-Widen D., Acevedo P. (2012). The status of tuberculosis in European wild mammals. Mammal Rev..

[22] Didkowska A., Orłowska B., Witkowski L., Olbrych K., Brzezińska S., Augustynowicz-Kopeć E., Krajewska-Wędzina M., Bereznowski A., Bielecki W., Krzysiak M. (2020). Biopsy and Tracheobronchial Aspirates as Additional Tools for the Diagnosis of Bovine Tuberculosis in Living European Bison (*Bison bonasus*). Animals.

[23] Orłowska B., Krajewska-Wędzina M., Augustynowicz-Kopeć E., Kozińska M., Brzezińska S., Zabost A., Didkowska A., Welz M., Kaczor S., Żmuda P. (2020). Epidemiological characterization of *Mycobacterium caprae* strains isolated from wildlife in the Bieszczady Mountains, on the border of Southeast Poland. BMC Vet. Res..

[24] Biek R., O’Hare A., Wright D., Mallon T., McCormick C., Orton R.J., McDowell S., Trewby H., Skuce R.A., Kao R.R. (2012). Whole genome sequencing reveals local transmission patterns of Mycobacterium bovis in sympatric cattle and badger populations. PLoS Pathogens..

[25] Akhmetova A., Guerrero J., McAdam P., Salvador L.C., Crispell J., Lavery J., Presho E., Kao R.R., Biek R., Menzies F. (2021). Genomic epidemiology of *Mycobacterium bovis* infection in sympatric badger and cattle populations in Northern Ireland. bioRxiv.

[26] Griffin J.M., Williams D.H., Kelly G.E., Clegg T.A., O’Boyle I., Collins J.D., More S.J. (2005). The impact of badger removal on the control of tuberculosis in cattle herds in Ireland. Prev. Vet. Med..

[27] Donnelly C.A., Woodroffe R., Cox D.R., Bourne F.J., Cheeseman C.L., Clifton-Hadley R.S., Wei G., Gettinby G., Gilks P., Jenkins H. (2006). Positive and negative effects of widespread badger culling on tuberculosis in cattle. Nature.

[28] Tabachnick W.J. (2010). Challenges in predicting climate and environmental effects on vector-borne disease episystems in a changing world. J. Exp. Biol..

[29] Murphy K.J., Morera-Pujol V., Ryan E., Byrne A.W., Breslin P., Ciuti S. (2022). Habitat availability alters the relative risk of a bovine tuberculosis breakdown in the aftermath of a commercial forest clearfell disturbance. J. Appl. Ecol..

[30] Martin S., O’Keeffe J., Byrne A., Rosen L., White P., McGrath G. (2020). Is moving from targeted culling to BCG-vaccination of badgers (*Meles meles*) associated with an unacceptable increased incidence of cattle herd tuberculosis in the Republic of Ireland? A practical non-inferiority wildlife intervention study in the Republic of Ireland (2011–2017). Prev. Vet. Med..

[31] More S.J. (2019). Can bovine TB be eradicated from the Republic of Ireland? Could this be achieved by 2030?. Ir. Vet. J..

[32] Byrne A.W., White P.W., McGrath G., Martin S.W. (2014). Risk of tuberculosis cattle herd breakdowns in Ireland: Effects of badger culling effort, density and historic large-scale interventions. Vet. Res..

[33] Campbell E.L., Byrne A.W., Menzies F.D., McBride K.R., McCormick C.M., Scantlebury M., Reid N. (2019). Interspecific visitation of cattle and badgers to fomites: A transmission risk for bovine tuberculosis?. Ecol. Evolution..

[34] Murphy D., Gormley E., Collins D.M., McGrath G., Sovsic E., Costello E., Corner L.A. (2011). Tuberculosis in cattle herds are sentinels for Mycobacterium bovis infection in European badgers (*Meles meles*): The Irish Greenfield Study. Vet. Microbiol..

[35] Byrne A.W., Kenny K., Fogarty U., O’keeffe J.J., More S.J., McGrath G., Teeling M., Martin S.W., Dohoo I.R. (2015). Spatial and temporal analyses of metrics of tuberculosis infection in badgers (*Meles meles*) from the Republic of Ireland: Trends in apparent prevalence. Prev. Vet. Med..

[36] Crispell J., Cassidy S., Kenny K., McGrath G., Warde S., Cameron H., Rossi G., MacWhite T., White P.C.L., Lycett S. (2020). Mycobacterium bovis genomics reveals transmission of infection between cattle and deer in Ireland. Microb. Genom..

[37] DAFM (2020). Forest Statistics Ireland. https://www.teagasc.ie/media/website/crops/forestry/advice/Forest-Statistics-Ireland-2020.pdf.

[38] DAFM (2019). Standards for Felling and Reforestation. https://www.teagasc.ie/media/website/crops/forestry/advice/Standards-for-Felling-and-Reforestation.pdf.

[39] Byrne A.W., Sleeman P.D., O’Keeffe J., Davenport J. (2012). The ecology of the European badger (*Meles meles*) in Ireland: A review. Biol. Environ. Proc. R. Irish Acad..

[40] Carden R.F., Carlin C.M., Marnell F., Mcelholm D., Hetherington J., Gammell M.P. (2011). Distribution and range expansion of deer in Ireland. Mammal Rev..

[41] Liu Y., Mccullagh A., Nieuwenhuis M. (2018). What factors affect national-scale deer population dynamics in the Republic of Ireland?. Scand. J. For. Res..

[42] Morera-Pujol V., Mostert P.S., Murphy K., Burkitt T., Coad B., McMahon B.J., Nieuwenhuis M., Morelle K., Ward A., Ciuti S. (2022). Bayesian species distribution models integrate presence-only and presence-absence data to predict deer distribution and relative abundance. bioRxiv.

[43] Potvin F., Bélanger L., Lowell K. (2000). Marten Habitat Selection in a Clearcut Boreal Landscape. Conserv. Biol..

[44] Fuller A.K., Harrison D.J., Lachowski H.J. (2004). Stand scale effects of partial harvesting and clearcutting on small mammals and forest structure. For. Ecol. Manag..

[45] Wäber K., Spencer J., Dolman P.M. (2013). Achieving landscape-scale deer management for biodiversity conservation: The need to consider sources and sinks. J. Wildl. Manag..

[46] Prentice J.C., Fox N.J., Hutchings M., White P.C.L., Davidson R., Marion G. (2019). When to kill a cull: Factors affecting the success of culling wildlife for disease control. J. R. Soc. Interface.

[47] Borremans B., Faust C., Manlove K.R., Sokolow S., Lloyd-Smith J.O. (2019). Cross-species pathogen spillover across ecosystem boundaries: Mechanisms and theory. Philos. Trans. R. Soc. B Biol. Sci..

[48] Barroso P., Breslin P., McGrath G., Madden J.M., Tratalos J.A., More S.J., Ryan E., Byrne A.W., Barrett D. (2022). Is there an association between road building and bovine tuberculosis herd risk? A three time-point study in Ireland, 2011–2019. Prev. Vet. Med..

[49] van Tonder A.J., Thornton M.J., Conlan A.J.K., Jolley K.A., Goolding L., Mitchell A.P., Dale J., Palkopoulou E., Hogarth P.J., Hewinson R.G. (2021). Inferring Mycobacterium bovis transmission between cattle and badgers using isolates from the Randomised Badger Culling Trial. PLOS Pathog..

[50] O’Corry-Crowe G., Eves J., Hayden T.J. (1993). Sett distribution, territory size and population density of badgers (*Meles meles* L.) in East Offaly. Badger.

[51] Riordan P., Delahay R.J., Cheeseman C., Johnson P.J., Macdonald D.W. (2011). Culling-Induced Changes in Badger (*Meles meles*) Behaviour, Social Organisation and the Epidemiology of Bovine Tuberculosis. PLoS ONE.

[52] Gaughran A., Mullen E., MacWhite T., Maher P., Kelly D.J., Kelly R., Good M., Marples N.M. (2021). Badger territoriality maintained despite disturbance of major road construction. PLoS ONE.

[53] Vial F., Donnelly C.A. (2012). Localized reactive badger culling increases risk of bovine tuberculosis in nearby cattle herds. Biol. Lett..

[54] O’Hagan M.J.H., Gordon A.W., McCormick C.M., Collins S.F., Trimble N.A., McGeown C.F., McHugh G.E., McBride K.R., Menzies F.D. (2021). Effect of selective removal of badgers (*Meles meles*) on ranging behaviour during a ‘Test and Vaccinate or Remove’ intervention in Northern Ireland. Epidemiol. Infect..

[55] Allen A.R., Milne G., McCormick C., Collins S., O’Hagan M., Skuce R., Trimble N., Harwood R., Menzies F., Byrne A.W. (2022). European badger (*Meles meles*) responses to low-intensity, selective culling: Using mark recapture and relatedness data to assess social perturbation. Ecol. Solut. Evid..

[56] Wright D.M., Reid N., Ian Montgomery W., Allen A.R., Skuce R.A., Kao R.R. (2015). Herd-level bovine tuberculosis risk factors: Assessing the role of low-level badger population disturbance. Sci. Rep..

[57] Walker J.G., Evans K.E., Vineer H.R., van Wyk J.A., Morgan E.R. (2018). Prediction and attenuation of seasonal spillover of parasites between wild and domestic ungulates in an arid mixed-use system. J. Appl. Ecol..

[58] Milne G., Byrne A.W., Campbell E., Graham J., McGrath J., Kirke R., McMaster W., Zimmermann J., Adenuga A.H. (2022). Quantifying Land Fragmentation in Northern Irish Cattle Enterprises. Land.

[59] Milne G., Graham J., McGrath J., Kirke R., McMaster W., Byrne A.W. (2022). Investigating Farm Fragmentation as a Risk Factor for Bovine Tuberculosis in Cattle Herds: A Matched Case-Control Study from Northern Ireland. Pathogens.

[60] Tsairidou S., Allen A., Banos G., Coffey M., Anacleto O., Byrne A.W., Skuce R.A., Glass E.J., Woolliams J.A., Doeschl-Wilson A.B. (2018). Can we breed cattle for lower bovine TB infectivity?. Front. Vet. Sci..

[61] Bernal J.L., Cummins S., Gasparrini A. (2017). Interrupted time series regression for the evaluation of public health interventions: A tutorial. Int. J. Epidemiol..

[62] Walker B., Sánchez-Vizcaíno F., Barker E.N. (2022). Effect of an antimicrobial stewardship intervention on the prescribing behaviours of companion animal veterinarians: A pre–post study. Vet. Rec..

[63] Buxton R.T., Lendrum P.E., Crooks K.R., Wittemyer G. (2018). Pairing camera traps and acoustic recorders to monitor the ecological impact of human disturbance. Glob. Ecol. Conserv..

[64] Sosa S., Sueur C., Puga-Gonzalez I. (2021). Network measures in animal social network analysis: Their strengths, limits, interpretations and uses. Methods Ecol. Evol..

[65] Abdou M., Frankena K., O’Keeffe J., Byrne A.W. (2016). Effect of culling and vaccination on bovine tuberculosis infection in a European badger (*Meles meles*) population by spatial simulation modelling. Prev. Vet. Med..

[66] Murphy K.J., Ciuti S., Kane A. (2020). An introduction to agent-based models as an accessible surrogate to field-based research and teaching. Ecol. Evol..

[67] Thiese M.S. (2014). Observational and interventional study design types; an overview. Biochem. Med..

[68] Lahuerta-Marin A., Milne M.G., McNair J., Skuce R.A., McBride S.H., Menzies F.D., McDowell S.J.W., Byrne A.W., Handel I.G., Bronsvoort B.D.C. (2018). Bayesian latent class estimation of sensitivity and specificity parameters of diagnostic tests for bovine tuberculosis in chronically infected herds in Northern Ireland. Vet. J..

[69] Byrne A.W., Quinn J.L., O’Keeffe J.J., Green S., Sleeman D.P., Martin S.W., Davenport J. (2014). Large-scale movements in European badgers: Has the tail of the movement kernel been underestimated?. J. Anim. Ecol..

[70] Byrne A.W., O’Keeffe J., Buesching C.D., Newman C. (2019). Push and pull factors driving movement in a social mammal: Context dependent behavioral plasticity at the landscape scale. Curr. Zool..

[71] More S.J., Clegg T.A., McGrath G., Collins J.D., Corner L.A.L., Gormley E. (2007). Does reactive badger culling lead to an increase in tuberculosis in cattle?. Vet. Rec..

[72] Giller P.S., Johnson M., O’Halloran J. (2002). Managing the Impacts of Forest Clearfelling on Stream Environments.

[73] Ramírez-Villaescusa A., Medley G., Mason S., Green L. (2009). Herd and individual animal risks associated with bovine tuberculosis skin test positivity in cattle in herds in south west England. Prev. Vet. Med..

[74] Burnham K.P., Anderson D.R., Huyvaert K.P. (2011). AIC model selection and multimodel inference in behavioral ecology: Some background, observations, and comparisons. Behav. Ecol. Sociobiol..

