# Supplementary material for: Disturbance Ecology Meets Bovine Tuberculosis (bTB) Epidemiology: A Before-and-After Study on the Association between Forest Clearfelling and bTB Herd Risk in Cattle Herds
Source: Pathogens. 2022 Jul 19;11(7):807. doi: 10.3390/pathogens11070807 (PMC9321662; doi:10.3390/pathogens11070807)
Supplement: Supplementary file 1 [file pathogens-11-00807-s001.zip › pathogens-1764736-supplementary.pdf]

Supplementary material

Table S1: Population averaged Generalised Estimating Equations model of the risk of disclosing 1 or more reactors post forest clearfelling relative to pre-clearfelling in Ireland 2015-2018

PARAMETER	OR	SE	Z	P	UPPER 95%CI	LOWER 95%CI
PRE/POST	1.187	0.068	3.010	0.003	1.062	1.328
LOG(HERD SIZE)	1.706	0.065	14.020	0.000	1.583	1.839
BEEF (REF)	1.000					
DAIRY	1.379	0.148	3.000	0.003	1.118	1.701
OTHER	0.513	0.094	-3.650	0.000	0.358	0.734
SUCKLER	1.008	0.092	0.090	0.930	0.843	1.205
PROPORTION FORESTRY	2.350	0.829	2.420	0.016	1.176	4.693
CARLOW	1.000					
CAVAN	1.968	0.542	2.460	0.014	1.146	3.377
CLARE	1.598	0.420	1.790	0.074	0.955	2.673
CORK	0.897	0.220	-0.450	0.656	0.555	1.449
DONEGAL	0.718	0.226	-1.050	0.294	0.388	1.331
GALWAY	1.303	0.353	0.980	0.329	0.766	2.217
KERRY	0.999	0.287	0.000	0.997	0.569	1.755
KILKENNY	0.783	0.215	-0.890	0.373	0.457	1.341
LAOIS	0.910	0.250	-0.340	0.732	0.531	1.560
LEITRIM	1.771	0.522	1.940	0.052	0.994	3.155
LIMERICK	0.676	0.226	-1.170	0.242	0.351	1.302
LONGFORD	0.472	0.171	-2.080	0.038	0.232	0.958
LOUTH	1.595	0.584	1.280	0.202	0.778	3.268
MAYO	0.861	0.253	-0.510	0.611	0.484	1.532
MEATH	1.581	0.505	1.430	0.151	0.846	2.955
MONAGHAN	1.695	0.516	1.730	0.083	0.933	3.079
OFFALY	0.943	0.281	-0.200	0.843	0.525	1.692
ROSCOMMON	0.944	0.288	-0.190	0.849	0.519	1.716
SLIGO	1.498	0.421	1.440	0.151	0.863	2.598
TIPPERARY	0.991	0.251	-0.030	0.972	0.604	1.627
WATERFORD	0.382	0.117	-3.150	0.002	0.210	0.695
WESTMEATH	1.791	0.500	2.090	0.037	1.037	3.094
WEXFORD	1.273	0.351	0.880	0.381	0.741	2.187
WICKLOW	3.217	0.791	4.750	0.000	1.987	5.208
CONSTANT	0.004	0.001	-19.640	0.000	0.002	0.006

>2 reactors model

Similar results were found when the breakdown threshold was raised to 2 or more reactors. However, the effect of pre/post clearfell was less certain, with the 95%ci of the odds ratio for the mixed effects model straddling 1 (0.992-1.419). This indicates that the positive association may not be robust when only 'large' breakdowns are used as our outcome variable. However, it should be noted, that the change of breakdown definition reduced the number of breakdown available to inform any trends, and therefore the power to detect an effect was diminished.

A similar result was found when the model was fit using a GEE framework; effect of pre/post OR: 1.17 (P=0.062; 95%ci: 0.992- 1.384).

Table S2: Mixed effect model with >2 reactors as breakdown threshold

PARAMETER	OR	SE	Z	P	UPPER 95%CI	LOWER 95%CI
PRE/POST	1.1865	0.1083	1.8700	0.0610	0.9922	1.4190
LOG(HERD SIZE)	2.3968	0.1576	13.3000	0.0000	2.1071	2.7264
BEEF (REF)						
DAIRY	1.5505	0.2705	2.5100	0.0120	1.1015	2.1825
OTHER	0.3778	0.1231	-2.9900	0.0030	0.1994	0.7156
SUCKLER	1.0879	0.1690	0.5400	0.5880	0.8022	1.4752
PROPORTION FORESTRY	5.2201	2.8598	3.0200	0.0030	1.7839	15.2756
CONSTANT	0.0002	0.0001	-24.7100	0.0000	0.0001	0.0003

Table S3: GEE model with >2 reactor threshold

PARAMETER	OR	SE	Z	P	UPPER 95%CI	LOWER 95%CI
PRE/POST	1.172	0.099	1.870	0.062	0.992	1.384
LOG(HERD SIZE)	2.274	0.137	13.640	0.000	2.021	2.559
BEEF (REF)						
DAIRY	1.523	0.247	2.590	0.009	1.108	2.093
OTHER	0.405	0.129	-2.850	0.004	0.217	0.755
SUCKLER	1.098	0.161	0.630	0.526	0.823	1.463

PROPORTION FORESTRY	5.001	2.488	3.240	0.001	1.886	13.258
CARLOW						
CAVAN	3.085	1.311	2.650	0.008	1.341	7.095
CLARE	1.998	0.833	1.660	0.097	0.882	4.525
CORK	1.131	0.438	0.320	0.751	0.529	2.417
DONEGAL	0.999	0.495	0.000	0.999	0.378	2.639
GALWAY	1.481	0.649	0.900	0.370	0.627	3.498
KERRY	1.377	0.621	0.710	0.478	0.569	3.332
KILKENNY	0.435	0.207	-1.750	0.081	0.171	1.107
LAOIS	0.845	0.375	-0.380	0.704	0.354	2.017
LEITRIM	2.520	1.195	1.950	0.051	0.994	6.385
LIMERICK	1.165	0.559	0.320	0.751	0.455	2.984
LONGFORD	0.319	0.224	-1.630	0.104	0.080	1.265
LOUTH	1.847	1.106	1.020	0.306	0.571	5.973
MAYO	0.986	0.480	-0.030	0.977	0.380	2.559
MEATH	2.386	1.134	1.830	0.067	0.940	6.057
MONAGHAN	3.015	1.361	2.440	0.015	1.245	7.304
OFFALY	1.079	0.507	0.160	0.871	0.430	2.710
ROSCOMMON	1.161	0.577	0.300	0.763	0.438	3.077
SLIGO	0.823	0.445	-0.360	0.719	0.285	2.375
TIPPERARY	1.191	0.473	0.440	0.661	0.546	2.596
WATERFORD	0.369	0.176	-2.090	0.037	0.145	0.942
WESTMEATH	2.963	1.252	2.570	0.010	1.294	6.784
WEXFORD	1.128	0.502	0.270	0.787	0.471	2.698
WICKLOW	4.638	1.789	3.980	0.000	2.177	9.879
CONSTANT	0.000	0.000	-17.210	0.000	0.000	0.001

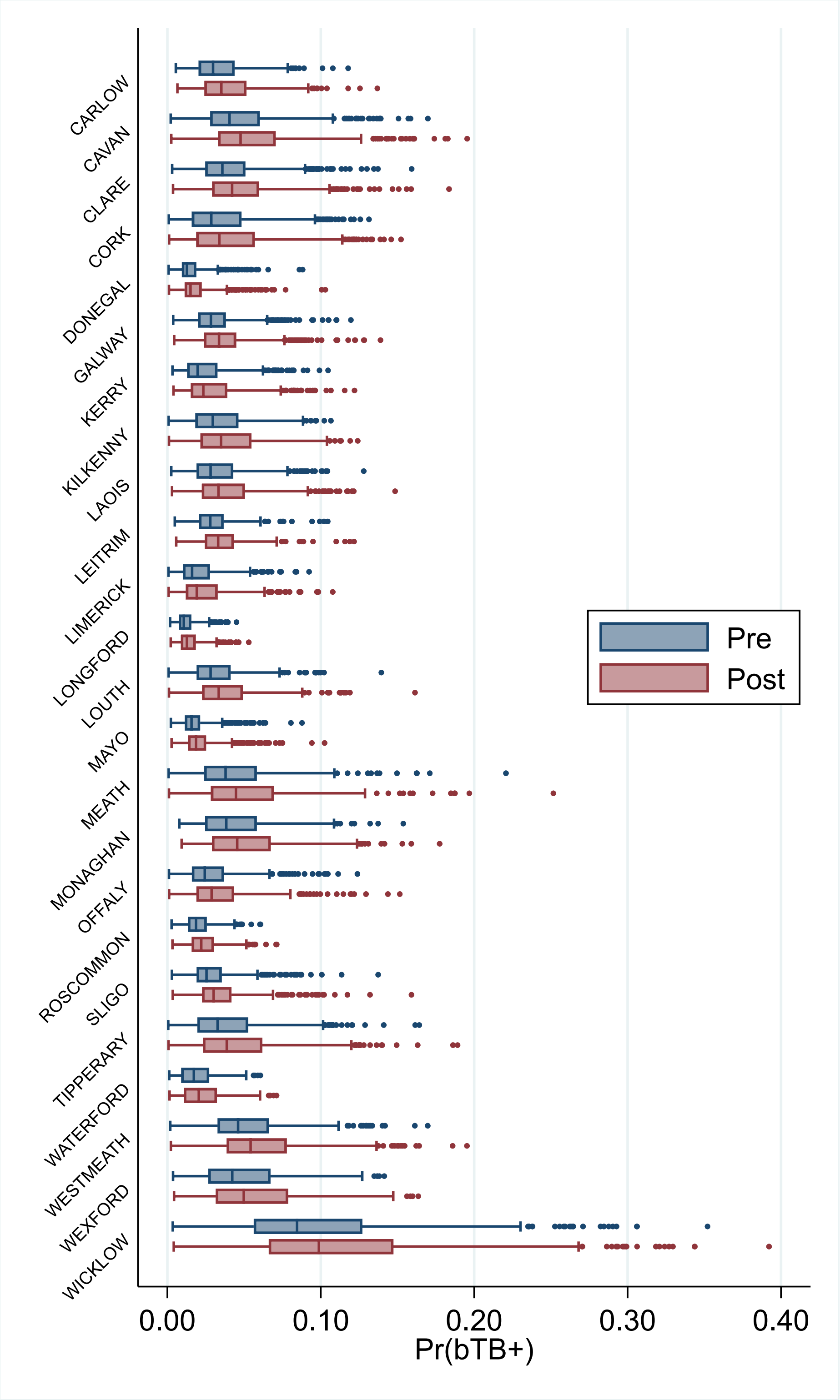

Figure S1: Variation in estimated risk of one or more bTB reactor being disclosed within cattle herds across counties prior-to and post a clearfelling forestry event in the vicinity (3km search radius)

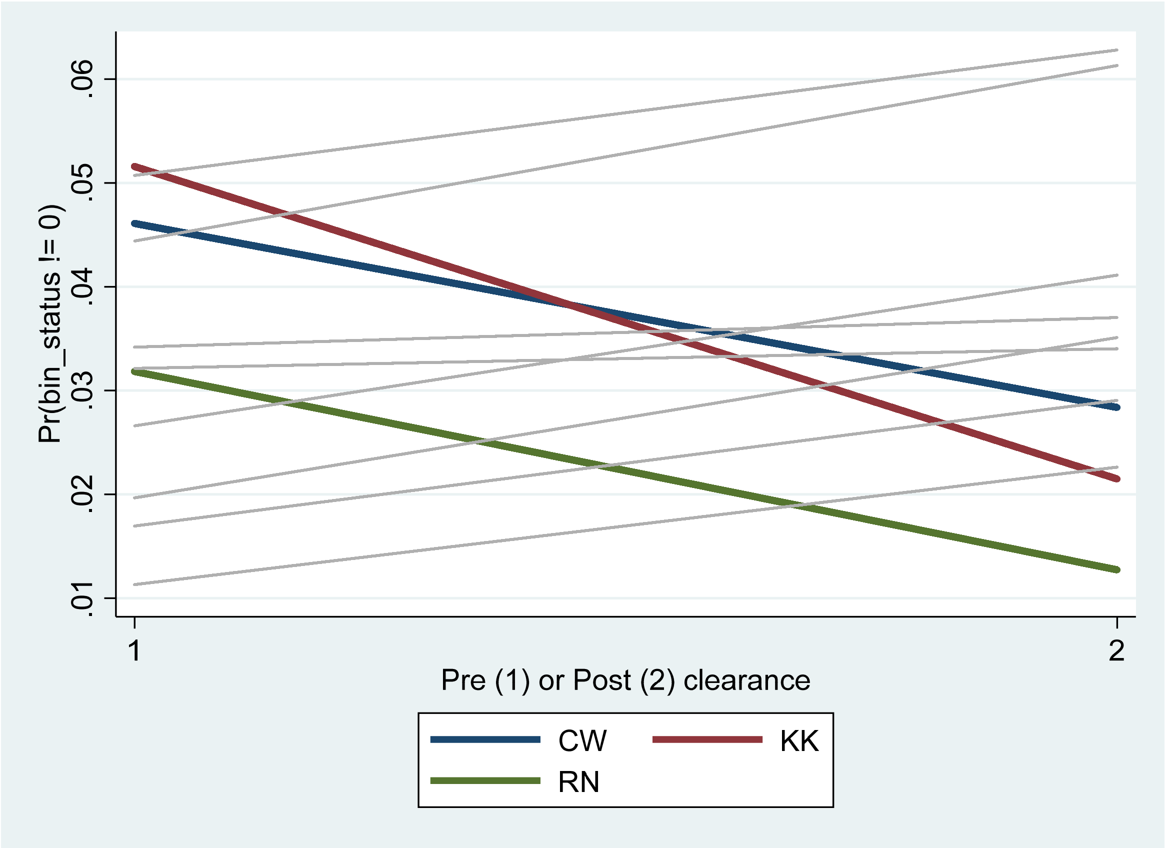

Figure S2: Relationship between bTB risk pre and post forest clearance activities estimated demonstrated 12 counties in Ireland. Three counties (Carlow = CW, Kilkenny = KK, and Roscommon = RN) had significantly different trend to all other counties modelled (total n=24).

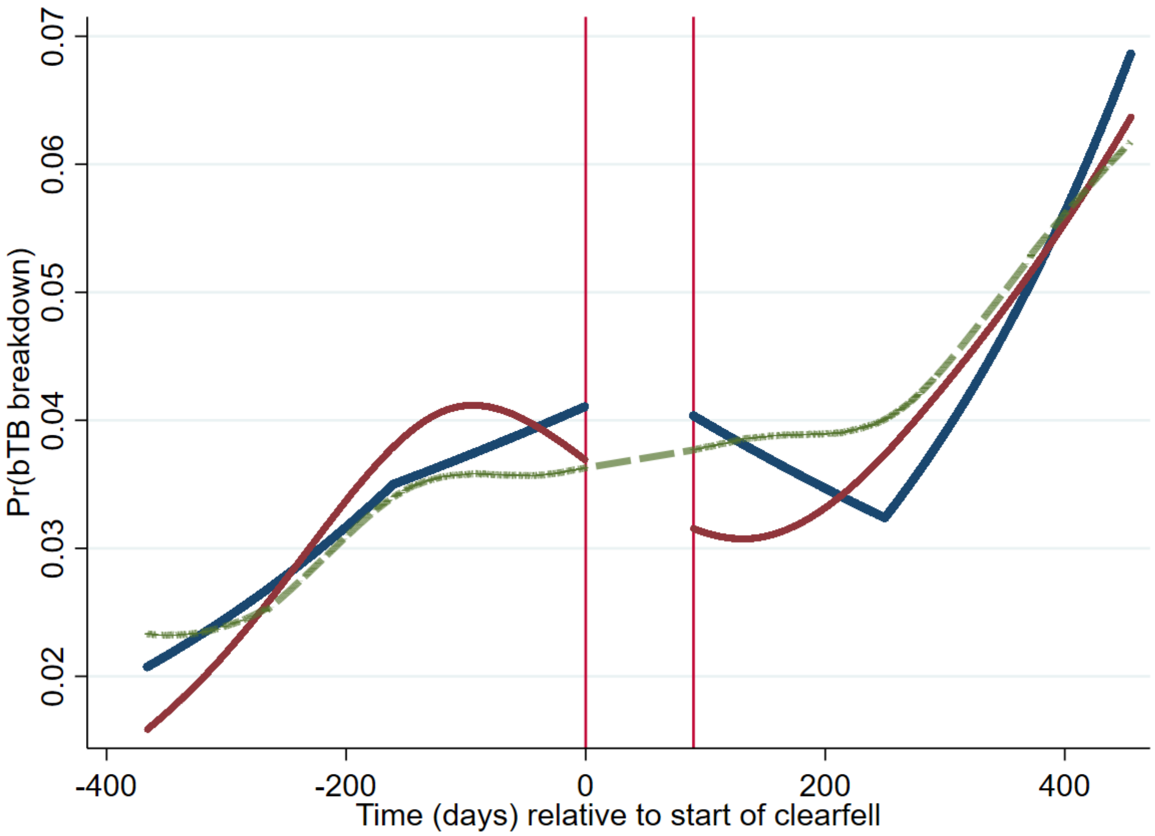

Figure S3: Time series plot of the estimated probability of bTB breakdown failure for cattle herd prior to (before day 0) and after a forest clearfell (after day 90). Dashed line (green) output from a locally weighted regression (LOWESS); red line is the mean predicted probability of failure from a cubic spline model; black line is the mean predicted probability of failure from a linear spline model.

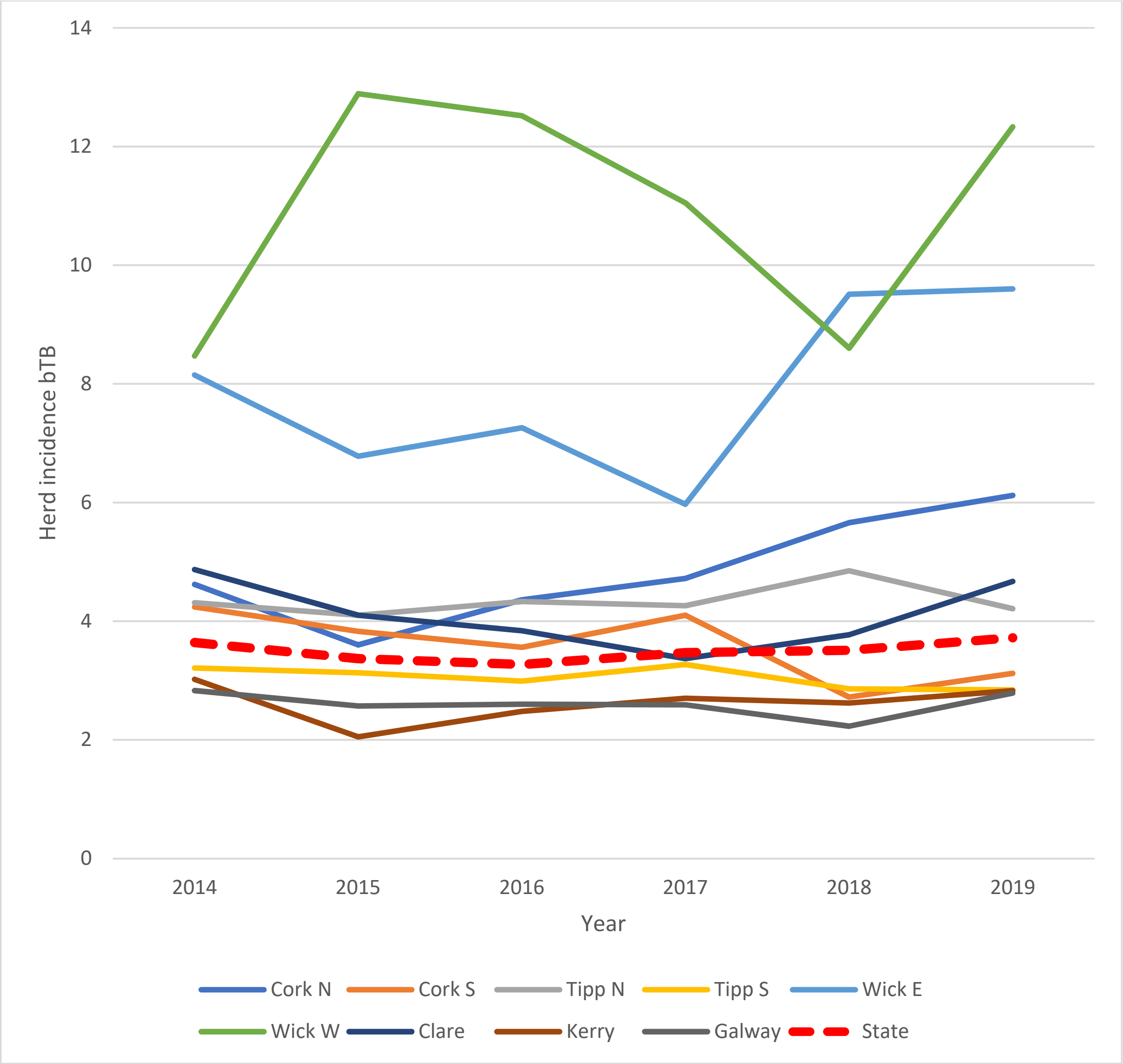

Figure S4: National trend in bTB herd incidence from year 2014-2019. The red dashed line is the overall country trend. Counties included were the most represented within the dataset with >3000 records each.

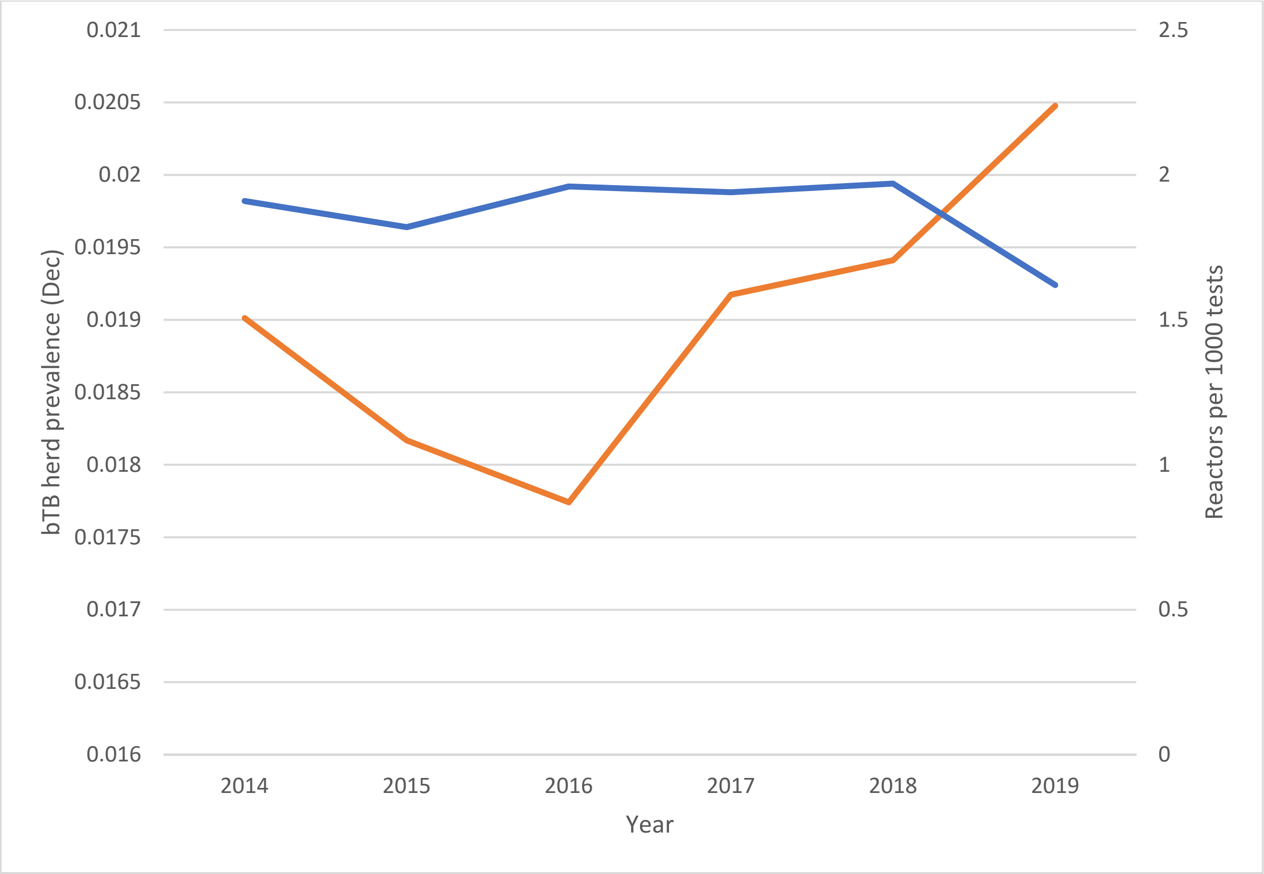

Figure S5: National trend in bTB herd prevalence (orange) and individual level test failures per 1000 tests (blue) from year 2014-2019.

Table S4: Example code and sample data (n=1000 herds; 2000 observations). Base univariable model: `xi: xtmelogit outcome_status i.pre_post ||herd_no:, or intpoints(12) level(95)`**, where outcome_status is whether the herd experienced a breakdown during the observation period; pre_post is a binary variable representing whether the obseration period was prior to or after a clearfell event; herd_no is an anonymised marker identifier variable for each herd.**

outcome_status	pre_post	herd_no
0	0	5001
0	1	5001
0	0	5002
0	1	5002
0	0	5003
0	1	5003
0	0	5004
0	1	5004
0	0	5005
0	1	5005
0	0	5006
0	1	5006
0	0	5007
0	1	5007
0	0	5008
0	1	5008
0	0	5009
0	1	5009
0	0	5010
0	1	5010
0	0	5011
0	1	5011
0	0	5012
0	1	5012
0	0	5013
0	1	5013
0	0	5014
0	1	5014
0	0	5015
0	1	5015
0	0	5016
0	1	5016
0	0	5017
0	1	5017
0	0	5018
0	1	5018
0	0	5019
0	1	5019
0	0	5020
0	1	5020
0	0	5021
0	1	5021
0	0	5022
0	1	5022
0	0	5023
0	1	5023
0	0	5024
0	1	5024
0	0	5025
0	1	5025
0	0	5026
0	1	5026
0	0	5027
0	1	5027
0	0	5028
0	1	5028
0	0	5029
0	1	5029
0	0	5030
0	1	5030
0	0	5031
0	1	5031
0	0	5032
0	1	5032
0	0	5033

0	1	5033
0	0	5034
0	1	5034
0	0	5035
0	1	5035
0	0	5036
0	1	5036
0	0	5037
0	1	5037
0	0	5038
0	1	5038
0	0	5039
0	1	5039
0	0	5040
0	1	5040
1	0	5041
0	1	5041
0	0	5042
0	1	5042
0	0	5043
0	1	5043
0	0	5044
0	1	5044
0	0	5045
0	1	5045
0	0	5046
0	1	5046
0	0	5047
0	1	5047
0	0	5048
1	1	5048
0	0	5049
0	1	5049
0	0	5050
0	1	5050
0	0	5051
0	1	5051
0	0	5052
0	1	5052
0	0	5053
0	1	5053
0	0	5054
0	1	5054
0	0	5055
0	1	5055
0	0	5056
0	1	5056
0	0	5057
0	1	5057
0	0	5058
0	1	5058
0	0	5059
0	1	5059
0	0	5060
0	1	5060
0	0	5061
0	1	5061
0	0	5062
0	1	5062
0	0	5063
0	1	5063
0	0	5064
1	1	5064
0	0	5065
0	1	5065
0	0	5066
1	1	5066
0	0	5067
1	1	5067
0	0	5068
0	1	5068
0	0	5069
0	1	5069
0	0	5070
0	1	5070
0	0	5071
0	1	5071

0	0	5072
1	1	5072
0	0	5073
0	1	5073
0	0	5074
0	1	5074
0	0	5075
0	1	5075
0	0	5076
0	1	5076
0	0	5077
0	1	5077
0	0	5078
0	1	5078
0	0	5079
0	1	5079
0	0	5080
0	1	5080
0	0	5081
0	1	5081
0	0	5082
0	1	5082
0	0	5083
0	1	5083
0	0	5084
0	1	5084
0	0	5085
0	1	5085
0	0	5086
1	1	5086
0	0	5087
0	1	5087
1	0	5088
0	1	5088
0	0	5089
0	1	5089
0	0	5090
0	1	5090
0	0	5091
0	1	5091
0	0	5092
0	1	5092
0	0	5093
0	1	5093
0	0	5094
1	1	5094
0	0	5095
0	1	5095
0	0	5096
0	1	5096
0	0	5097
0	1	5097
0	0	5098
0	1	5098
0	0	5099
0	1	5099
0	0	5100
0	1	5100
0	0	5101
0	1	5101
0	0	5102
0	1	5102
0	0	5103
0	1	5103
0	0	5104
0	1	5104
0	0	5105
0	1	5105
0	0	5106
0	1	5106
0	0	5107
0	1	5107
0	0	5108
0	1	5108
0	0	5109
0	1	5109
1	0	5110

0	1	5110
0	0	5111
0	1	5111
0	0	5112
0	1	5112
0	0	5113
0	1	5113
0	0	5114
0	1	5114
0	0	5115
0	1	5115
0	0	5116
0	1	5116
0	0	5117
0	1	5117
0	0	5118
0	1	5118
0	0	5119
0	1	5119
0	0	5120
0	1	5120
0	0	5121
0	1	5121
0	0	5122
0	1	5122
0	0	5123
0	1	5123
0	0	5124
0	1	5124
0	0	5125
0	1	5125
0	0	5126
0	1	5126
0	0	5127
0	1	5127
0	0	5128
0	1	5128
0	0	5129
0	1	5129
0	0	5130
0	1	5130
0	0	5131
0	1	5131
0	0	5132
0	1	5132
0	0	5133
0	1	5133
0	0	5134
0	1	5134
0	0	5135
0	1	5135
0	0	5136
0	1	5136
0	0	5137
0	1	5137
0	0	5138
0	1	5138
0	0	5139
0	1	5139
0	0	5140
0	1	5140
0	0	5141
0	1	5141
0	0	5142
0	1	5142
0	0	5143
0	1	5143
0	0	5144
0	1	5144
0	0	5145
0	1	5145
0	0	5146
0	1	5146
0	0	5147
0	1	5147
0	0	5148
0	1	5148

0	0	5149
0	1	5149
0	0	5150
0	1	5150
0	0	5151
0	1	5151
0	0	5152
0	1	5152
0	0	5153
0	1	5153
0	0	5154
0	1	5154
0	0	5155
0	1	5155
0	0	5156
0	1	5156
0	0	5157
0	1	5157
0	0	5158
0	1	5158
0	0	5159
0	1	5159
0	0	5160
0	1	5160
1	0	5161
0	1	5161
0	0	5162
0	1	5162
0	0	5163
0	1	5163
0	0	5164
0	1	5164
0	0	5165
0	1	5165
0	0	5166
0	1	5166
0	0	5167
0	1	5167
0	0	5168
0	1	5168
0	0	5169
0	1	5169
0	0	5170
0	1	5170
0	0	5171
0	1	5171
0	0	5172
0	1	5172
0	0	5173
0	1	5173
0	0	5174
0	1	5174
0	0	5175
0	1	5175
0	0	5176
0	1	5176
0	0	5177
0	1	5177
0	0	5178
1	1	5178
0	0	5179
0	1	5179
0	0	5180
0	1	5180
0	0	5181
0	1	5181
0	0	5182
0	1	5182
0	0	5183
0	1	5183
1	0	5184
0	1	5184
0	0	5185
0	1	5185
0	0	5186
0	1	5186
0	0	5187

0	1	5187
1	0	5188
0	1	5188
0	0	5189
0	1	5189
0	0	5190
0	1	5190
1	0	5191
0	1	5191
0	0	5192
0	1	5192
1	0	5193
0	1	5193
0	0	5194
0	1	5194
0	0	5195
1	1	5195
0	0	5196
0	1	5196
0	0	5197
0	1	5197
0	0	5198
0	1	5198
0	0	5199
0	1	5199
0	0	5200
0	1	5200
0	0	5201
0	1	5201
0	0	5202
0	1	5202
0	0	5203
0	1	5203
0	0	5204
0	1	5204
0	0	5205
0	1	5205
1	0	5206
0	1	5206
0	0	5207
0	1	5207
0	0	5208
1	1	5208
0	0	5209
0	1	5209
0	0	5210
0	1	5210
0	0	5211
0	1	5211
0	0	5212
0	1	5212
0	0	5213
0	1	5213
0	0	5214
0	1	5214
0	0	5215
0	1	5215
0	0	5216
0	1	5216
0	0	5217
0	1	5217
0	0	5218
0	1	5218
0	0	5219
0	1	5219
0	0	5220
0	1	5220
0	0	5221
0	1	5221
0	0	5222
0	1	5222
0	0	5223
0	1	5223
0	0	5224
0	1	5224
0	0	5225
0	1	5225

0	0	5226
0	1	5226
0	0	5227
0	1	5227
0	0	5228
0	1	5228
0	0	5229
0	1	5229
0	0	5230
0	1	5230
0	0	5231
0	1	5231
0	0	5232
0	1	5232
0	0	5233
0	1	5233
0	0	5234
0	1	5234
0	0	5235
0	1	5235
0	0	5236
0	1	5236
0	0	5237
0	1	5237
0	0	5238
0	1	5238
0	0	5239
0	1	5239
0	0	5240
0	1	5240
0	0	5241
0	1	5241
0	0	5242
0	1	5242
0	0	5243
0	1	5243
0	0	5244
0	1	5244
0	0	5245
0	1	5245
0	0	5246
0	1	5246
0	0	5247
0	1	5247
0	0	5248
0	1	5248
0	0	5249
0	1	5249
0	0	5250
0	1	5250
0	0	5251
0	1	5251
0	0	5252
0	1	5252
0	0	5253
0	1	5253
0	0	5254
0	1	5254
0	0	5255
0	1	5255
0	0	5256
0	1	5256
0	0	5257
0	1	5257
0	0	5258
0	1	5258
0	0	5259
0	1	5259
0	0	5260
0	1	5260
0	0	5261
0	1	5261
0	0	5262
0	1	5262
0	0	5263
0	1	5263
0	0	5264

0	1	5264
0	0	5265
0	1	5265
0	0	5266
0	1	5266
0	0	5267
0	1	5267
0	0	5268
0	1	5268
0	0	5269
0	1	5269
0	0	5270
0	1	5270
0	0	5271
0	1	5271
0	0	5272
0	1	5272
0	0	5273
0	1	5273
0	0	5274
0	1	5274
0	0	5275
0	1	5275
0	0	5276
0	1	5276
0	0	5277
0	1	5277
0	0	5278
0	1	5278
0	0	5279
0	1	5279
0	0	5280
0	1	5280
0	0	5281
0	1	5281
0	0	5282
0	1	5282
0	0	5283
0	1	5283
0	0	5284
0	1	5284
0	0	5285
0	1	5285
0	0	5286
0	1	5286
0	0	5287
0	1	5287
0	0	5288
0	1	5288
0	0	5289
0	1	5289
0	0	5290
0	1	5290
0	0	5291
0	1	5291
0	0	5292
0	1	5292
0	0	5293
0	1	5293
0	0	5294
0	1	5294
0	0	5295
0	1	5295
0	0	5296
0	1	5296
0	0	5297
0	1	5297
0	0	5298
0	1	5298
0	0	5299
0	1	5299
0	0	5300
0	1	5300
0	0	5301
0	1	5301
0	0	5302
0	1	5302

0	0	5303
0	1	5303
0	0	5304
0	1	5304
0	0	5305
0	1	5305
0	0	5306
0	1	5306
0	0	5307
0	1	5307
0	0	5308
0	1	5308
0	0	5309
0	1	5309
0	0	5310
0	1	5310
0	0	5311
0	1	5311
0	0	5312
0	1	5312
0	0	5313
0	1	5313
0	0	5314
0	1	5314
0	0	5315
0	1	5315
0	0	5316
0	1	5316
0	0	5317
0	1	5317
0	0	5318
0	1	5318
0	0	5319
0	1	5319
0	0	5320
0	1	5320
0	0	5321
0	1	5321
0	0	5322
0	1	5322
0	0	5323
0	1	5323
0	0	5324
0	1	5324
0	0	5325
0	1	5325
0	0	5326
0	1	5326
0	0	5327
0	1	5327
0	0	5328
0	1	5328
0	0	5329
0	1	5329
0	0	5330
0	1	5330
0	0	5331
0	1	5331
0	0	5332
0	1	5332
0	0	5333
1	1	5333
0	0	5334
0	1	5334
1	0	5335
0	1	5335
0	0	5336
0	1	5336
0	0	5337
0	1	5337
0	0	5338
0	1	5338
0	0	5339
0	1	5339
0	0	5340
0	1	5340
0	0	5341

0	1	5341
0	0	5342
0	1	5342
0	0	5343
0	1	5343
0	0	5344
0	1	5344
0	0	5345
0	1	5345
0	0	5346
0	1	5346
0	0	5347
0	1	5347
0	0	5348
0	1	5348
0	0	5349
0	1	5349
0	0	5350
0	1	5350
0	0	5351
0	1	5351
0	0	5352
0	1	5352
0	0	5353
0	1	5353
0	0	5354
0	1	5354
0	0	5355
0	1	5355
0	0	5356
0	1	5356
0	0	5357
0	1	5357
0	0	5358
0	1	5358
0	0	5359
0	1	5359
0	0	5360
0	1	5360
0	0	5361
0	1	5361
0	0	5362
0	1	5362
0	0	5363
1	1	5363
0	0	5364
0	1	5364
0	0	5365
0	1	5365
0	0	5366
1	1	5366
0	0	5367
0	1	5367
0	0	5368
0	1	5368
0	0	5369
0	1	5369
0	0	5370
0	1	5370
0	0	5371
0	1	5371
0	0	5372
0	1	5372
0	0	5373
0	1	5373
0	0	5374
0	1	5374
0	0	5375
0	1	5375
0	0	5376
1	1	5376
0	0	5377
0	1	5377
0	0	5378
0	1	5378
1	0	5379
0	1	5379

0	0	5380
0	1	5380
0	0	5381
0	1	5381
0	0	5382
1	1	5382
0	0	5383
0	1	5383
0	0	5384
0	1	5384
0	0	5385
0	1	5385
0	0	5386
0	1	5386
0	0	5387
0	1	5387
0	0	5388
0	1	5388
0	0	5389
0	1	5389
0	0	5390
0	1	5390
0	0	5391
0	1	5391
0	0	5392
0	1	5392
0	0	5393
1	1	5393
0	0	5394
0	1	5394
0	0	5395
1	1	5395
0	0	5396
0	1	5396
0	0	5397
0	1	5397
0	0	5398
0	1	5398
0	0	5399
0	1	5399
0	0	5400
1	1	5400
0	0	5401
0	1	5401
0	0	5402
0	1	5402
0	0	5403
0	1	5403
0	0	5404
0	1	5404
0	0	5405
0	1	5405
0	0	5406
0	1	5406
0	0	5407
0	1	5407
0	0	5408
0	1	5408
0	0	5409
0	1	5409
0	0	5410
0	1	5410
0	0	5411
0	1	5411
0	0	5412
0	1	5412
0	0	5413
0	1	5413
0	0	5414
0	1	5414
0	0	5415
0	1	5415
0	0	5416
0	1	5416
0	0	5417
0	1	5417
0	0	5418

0	1	5418
0	0	5419
0	1	5419
0	0	5420
0	1	5420
0	0	5421
0	1	5421
0	0	5422
0	1	5422
0	0	5423
0	1	5423
0	0	5424
0	1	5424
0	0	5425
0	1	5425
0	0	5426
0	1	5426
0	0	5427
0	1	5427
0	0	5428
0	1	5428
0	0	5429
0	1	5429
0	0	5430
0	1	5430
0	0	5431
0	1	5431
0	0	5432
0	1	5432
0	0	5433
0	1	5433
0	0	5434
0	1	5434
0	0	5435
0	1	5435
0	0	5436
1	1	5436
1	0	5437
0	1	5437
0	0	5438
0	1	5438
0	0	5439
0	1	5439
0	0	5440
0	1	5440
0	0	5441
0	1	5441
0	0	5442
0	1	5442
0	0	5443
0	1	5443
0	0	5444
0	1	5444
0	0	5445
0	1	5445
0	0	5446
0	1	5446
0	0	5447
0	1	5447
0	0	5448
0	1	5448
0	0	5449
0	1	5449
0	0	5450
0	1	5450
0	0	5451
0	1	5451
0	0	5452
0	1	5452
0	0	5453
0	1	5453
0	0	5454
0	1	5454
0	0	5455
0	1	5455
0	0	5456
0	1	5456

0	0	5457
0	1	5457
0	0	5458
0	1	5458
0	0	5459
0	1	5459
0	0	5460
0	1	5460
0	0	5461
1	1	5461
0	0	5462
0	1	5462
0	0	5463
0	1	5463
0	0	5464
0	1	5464
0	0	5465
0	1	5465
0	0	5466
1	1	5466
0	0	5467
0	1	5467
0	0	5468
0	1	5468
0	0	5469
0	1	5469
0	0	5470
0	1	5470
0	0	5471
0	1	5471
0	0	5472
0	1	5472
0	0	5473
0	1	5473
0	0	5474
0	1	5474
0	0	5475
0	1	5475
0	0	5476
0	1	5476
0	0	5477
0	1	5477
0	0	5478
0	1	5478
0	0	5479
0	1	5479
0	0	5480
0	1	5480
0	0	5481
0	1	5481
0	0	5482
0	1	5482
0	0	5483
1	1	5483
0	0	5484
0	1	5484
0	0	5485
0	1	5485
0	0	5486
0	1	5486
0	0	5487
0	1	5487
0	0	5488
0	1	5488
0	0	5489
0	1	5489
0	0	5490
0	1	5490
0	0	5491
0	1	5491
0	0	5492
0	1	5492
0	0	5493
0	1	5493
0	0	5494
0	1	5494
0	0	5495

0	1	5495
0	0	5496
0	1	5496
0	0	5497
0	1	5497
0	0	5498
0	1	5498
0	0	5499
0	1	5499
0	0	5500
0	1	5500
0	0	5501
0	1	5501
0	0	5502
0	1	5502
0	0	5503
0	1	5503
0	0	5504
0	1	5504
0	0	5505
0	1	5505
0	0	5506
0	1	5506
0	0	5507
0	1	5507
0	0	5508
0	1	5508
0	0	5509
0	1	5509
0	0	5510
0	1	5510
0	0	5511
0	1	5511
0	0	5512
0	1	5512
0	0	5513
0	1	5513
0	0	5514
0	1	5514
0	0	5515
0	1	5515
0	0	5516
0	1	5516
0	0	5517
0	1	5517
0	0	5518
0	1	5518
0	0	5519
0	1	5519
0	0	5520
0	1	5520
0	0	5521
0	1	5521
0	0	5522
0	1	5522
0	0	5523
0	1	5523
0	0	5524
0	1	5524
0	0	5525
0	1	5525
0	0	5526
0	1	5526
0	0	5527
0	1	5527
0	0	5528
0	1	5528
0	0	5529
0	1	5529
0	0	5530
0	1	5530
0	0	5531
0	1	5531
0	0	5532
0	1	5532
0	0	5533
0	1	5533

0	0	5534
0	1	5534
0	0	5535
0	1	5535
0	0	5536
0	1	5536
0	0	5537
0	1	5537
0	0	5538
0	1	5538
0	0	5539
0	1	5539
0	0	5540
0	1	5540
0	0	5541
0	1	5541
0	0	5542
0	1	5542
0	0	5543
0	1	5543
0	0	5544
0	1	5544
0	0	5545
0	1	5545
1	0	5546
0	1	5546
0	0	5547
1	1	5547
0	0	5548
0	1	5548
0	0	5549
0	1	5549
0	0	5550
0	1	5550
0	0	5551
0	1	5551
0	0	5552
0	1	5552
0	0	5553
0	1	5553
0	0	5554
0	1	5554
0	0	5555
0	1	5555
0	0	5556
0	1	5556
0	0	5557
0	1	5557
0	0	5558
0	1	5558
0	0	5559
0	1	5559
0	0	5560
0	1	5560
0	0	5561
0	1	5561
0	0	5562
0	1	5562
0	0	5563
0	1	5563
1	0	5564
0	1	5564
0	0	5565
0	1	5565
0	0	5566
0	1	5566
0	0	5567
0	1	5567
0	0	5568
0	1	5568
0	0	5569
0	1	5569
0	0	5570
0	1	5570
0	0	5571
0	1	5571
0	0	5572

0	1	5572
0	0	5573
0	1	5573
0	0	5574
0	1	5574
0	0	5575
0	1	5575
0	0	5576
0	1	5576
0	0	5577
0	1	5577
0	0	5578
0	1	5578
0	0	5579
0	1	5579
0	0	5580
0	1	5580
0	0	5581
0	1	5581
0	0	5582
0	1	5582
0	0	5583
0	1	5583
0	0	5584
0	1	5584
0	0	5585
1	1	5585
0	0	5586
0	1	5586
0	0	5587
0	1	5587
0	0	5588
0	1	5588
0	0	5589
0	1	5589
0	0	5590
0	1	5590
0	0	5591
0	1	5591
0	0	5592
0	1	5592
0	0	5593
0	1	5593
0	0	5594
0	1	5594
0	0	5595
0	1	5595
0	0	5596
0	1	5596
0	0	5597
0	1	5597
0	0	5598
0	1	5598
0	0	5599
0	1	5599
0	0	5600
0	1	5600
0	0	5601
0	1	5601
0	0	5602
0	1	5602
0	0	5603
0	1	5603
0	0	5604
0	1	5604
0	0	5605
0	1	5605
0	0	5606
0	1	5606
0	0	5607
0	1	5607
0	0	5608
0	1	5608
0	0	5609
0	1	5609
0	0	5610
0	1	5610

0	0	5611
0	1	5611
0	0	5612
0	1	5612
0	0	5613
0	1	5613
0	0	5614
0	1	5614
0	0	5615
0	1	5615
0	0	5616
0	1	5616
0	0	5617
0	1	5617
0	0	5618
0	1	5618
0	0	5619
0	1	5619
0	0	5620
0	1	5620
0	0	5621
0	1	5621
0	0	5622
0	1	5622
0	0	5623
0	1	5623
1	0	5624
0	1	5624
0	0	5625
0	1	5625
0	0	5626
0	1	5626
0	0	5627
0	1	5627
0	0	5628
0	1	5628
0	0	5629
0	1	5629
0	0	5630
0	1	5630
0	0	5631
0	1	5631
0	0	5632
0	1	5632
0	0	5633
0	1	5633
0	0	5634
0	1	5634
0	0	5635
0	1	5635
1	0	5636
1	1	5636
0	0	5637
0	1	5637
0	0	5638
0	1	5638
0	0	5639
0	1	5639
0	0	5640
0	1	5640
0	0	5641
0	1	5641
0	0	5642
0	1	5642
0	0	5643
0	1	5643
0	0	5644
0	1	5644
0	0	5645
0	1	5645
0	0	5646
0	1	5646
0	0	5647
0	1	5647
0	0	5648
0	1	5648
0	0	5649

0	1	5649
0	0	5650
0	1	5650
0	0	5651
0	1	5651
0	0	5652
0	1	5652
0	0	5653
0	1	5653
1	0	5654
0	1	5654
0	0	5655
0	1	5655
0	0	5656
0	1	5656
0	0	5657
1	1	5657
0	0	5658
1	1	5658
0	0	5659
0	1	5659
0	0	5660
0	1	5660
0	0	5661
0	1	5661
0	0	5662
0	1	5662
0	0	5663
0	1	5663
0	0	5664
0	1	5664
0	0	5665
0	1	5665
0	0	5666
0	1	5666
1	0	5667
1	1	5667
0	0	5668
0	1	5668
0	0	5669
1	1	5669
0	0	5670
0	1	5670
0	0	5671
0	1	5671
0	0	5672
0	1	5672
0	0	5673
0	1	5673
0	0	5674
0	1	5674
1	0	5675
0	1	5675
1	0	5676
1	1	5676
0	0	5677
0	1	5677
1	0	5678
0	1	5678
0	0	5679
0	1	5679
0	0	5680
0	1	5680
0	0	5681
0	1	5681
0	0	5682
0	1	5682
0	0	5683
0	1	5683
0	0	5684
0	1	5684
0	0	5685
0	1	5685
0	0	5686
0	1	5686
0	0	5687
0	1	5687

0	0	5688
0	1	5688
0	0	5689
0	1	5689
0	0	5690
0	1	5690
0	0	5691
0	1	5691
0	0	5692
0	1	5692
0	0	5693
0	1	5693
0	0	5694
0	1	5694
0	0	5695
0	1	5695
0	0	5696
0	1	5696
1	0	5697
0	1	5697
0	0	5698
0	1	5698
0	0	5699
0	1	5699
0	0	5700
0	1	5700
0	0	5701
0	1	5701
0	0	5702
0	1	5702
0	0	5703
0	1	5703
0	0	5704
0	1	5704
0	0	5705
0	1	5705
0	0	5706
0	1	5706
0	0	5707
0	1	5707
0	0	5708
0	1	5708
0	0	5709
0	1	5709
0	0	5710
0	1	5710
0	0	5711
0	1	5711
0	0	5712
0	1	5712
0	0	5713
0	1	5713
0	0	5714
0	1	5714
0	0	5715
0	1	5715
0	0	5716
0	1	5716
0	0	5717
0	1	5717
0	0	5718
0	1	5718
0	0	5719
0	1	5719
0	0	5720
0	1	5720
0	0	5721
0	1	5721
0	0	5722
0	1	5722
0	0	5723
0	1	5723
0	0	5724
0	1	5724
0	0	5725
0	1	5725
0	0	5726

0	1	5726
0	0	5727
0	1	5727
0	0	5728
0	1	5728
0	0	5729
0	1	5729
0	0	5730
0	1	5730
0	0	5731
0	1	5731
0	0	5732
0	1	5732
0	0	5733
0	1	5733
0	0	5734
0	1	5734
0	0	5735
0	1	5735
0	0	5736
0	1	5736
0	0	5737
0	1	5737
0	0	5738
0	1	5738
0	0	5739
0	1	5739
0	0	5740
0	1	5740
0	0	5741
0	1	5741
0	0	5742
0	1	5742
0	0	5743
0	1	5743
0	0	5744
0	1	5744
0	0	5745
1	1	5745
0	0	5746
0	1	5746
0	0	5747
0	1	5747
0	0	5748
0	1	5748
0	0	5749
0	1	5749
0	0	5750
0	1	5750
0	0	5751
0	1	5751
0	0	5752
0	1	5752
0	0	5753
0	1	5753
0	0	5754
0	1	5754
0	0	5755
0	1	5755
0	0	5756
0	1	5756
0	0	5757
0	1	5757
0	0	5758
0	1	5758
0	0	5759
0	1	5759
0	0	5760
0	1	5760
0	0	5761
0	1	5761
0	0	5762
0	1	5762
0	0	5763
0	1	5763
0	0	5764
0	1	5764

0	0	5765
0	1	5765
0	0	5766
0	1	5766
0	0	5767
0	1	5767
0	0	5768
0	1	5768
0	0	5769
0	1	5769
0	0	5770
0	1	5770
0	0	5771
0	1	5771
0	0	5772
0	1	5772
0	0	5773
0	1	5773
0	0	5774
0	1	5774
0	0	5775
0	1	5775
0	0	5776
0	1	5776
0	0	5777
0	1	5777
0	0	5778
0	1	5778
0	0	5779
0	1	5779
0	0	5780
0	1	5780
0	0	5781
0	1	5781
0	0	5782
0	1	5782
0	0	5783
0	1	5783
0	0	5784
0	1	5784
0	0	5785
0	1	5785
1	0	5786
0	1	5786
0	0	5787
0	1	5787
0	0	5788
0	1	5788
0	0	5789
0	1	5789
0	0	5790
0	1	5790
0	0	5791
0	1	5791
0	0	5792
0	1	5792
0	0	5793
0	1	5793
0	0	5794
0	1	5794
0	0	5795
0	1	5795
0	0	5796
0	1	5796
0	0	5797
0	1	5797
0	0	5798
0	1	5798
0	0	5799
0	1	5799
0	0	5800
0	1	5800
0	0	5801
0	1	5801
0	0	5802
0	1	5802
0	0	5803

0	1	5803
0	0	5804
0	1	5804
0	0	5805
0	1	5805
0	0	5806
0	1	5806
0	0	5807
0	1	5807
0	0	5808
0	1	5808
0	0	5809
0	1	5809
0	0	5810
0	1	5810
0	0	5811
0	1	5811
0	0	5812
0	1	5812
0	0	5813
0	1	5813
0	0	5814
0	1	5814
0	0	5815
0	1	5815
0	0	5816
0	1	5816
0	0	5817
0	1	5817
0	0	5818
0	1	5818
0	0	5819
0	1	5819
0	0	5820
0	1	5820
0	0	5821
1	1	5821
0	0	5822
1	1	5822
0	0	5823
0	1	5823
0	0	5824
0	1	5824
0	0	5825
0	1	5825
0	0	5826
1	1	5826
0	0	5827
0	1	5827
0	0	5828
0	1	5828
0	0	5829
0	1	5829
0	0	5830
0	1	5830
0	0	5831
0	1	5831
0	0	5832
0	1	5832
0	0	5833
0	1	5833
0	0	5834
0	1	5834
0	0	5835
0	1	5835
0	0	5836
0	1	5836
0	0	5837
0	1	5837
0	0	5838
0	1	5838
0	0	5839
0	1	5839
0	0	5840
0	1	5840
0	0	5841
0	1	5841

0	0	5842
0	1	5842
0	0	5843
0	1	5843
0	0	5844
0	1	5844
0	0	5845
0	1	5845
0	0	5846
0	1	5846
0	0	5847
0	1	5847
0	0	5848
0	1	5848
0	0	5849
0	1	5849
0	0	5850
0	1	5850
0	0	5851
0	1	5851
0	0	5852
0	1	5852
0	0	5853
0	1	5853
0	0	5854
0	1	5854
0	0	5855
0	1	5855
0	0	5856
0	1	5856
0	0	5857
0	1	5857
0	0	5858
0	1	5858
0	0	5859
0	1	5859
0	0	5860
0	1	5860
0	0	5861
0	1	5861
0	0	5862
0	1	5862
0	0	5863
1	1	5863
1	0	5864
0	1	5864
0	0	5865
0	1	5865
0	0	5866
0	1	5866
0	0	5867
0	1	5867
0	0	5868
0	1	5868
0	0	5869
0	1	5869
0	0	5870
0	1	5870
0	0	5871
0	1	5871
0	0	5872
0	1	5872
0	0	5873
0	1	5873
0	0	5874
0	1	5874
0	0	5875
0	1	5875
0	0	5876
0	1	5876
0	0	5877
0	1	5877
0	0	5878
0	1	5878
0	0	5879
0	1	5879
0	0	5880

0	1	5880
0	0	5881
0	1	5881
0	0	5882
0	1	5882
0	0	5883
0	1	5883
0	0	5884
0	1	5884
0	0	5885
0	1	5885
0	0	5886
0	1	5886
0	0	5887
0	1	5887
0	0	5888
0	1	5888
0	0	5889
0	1	5889
0	0	5890
0	1	5890
0	0	5891
0	1	5891
0	0	5892
0	1	5892
0	0	5893
0	1	5893
0	0	5894
0	1	5894
0	0	5895
0	1	5895
0	0	5896
0	1	5896
0	0	5897
0	1	5897
0	0	5898
0	1	5898
0	0	5899
0	1	5899
0	0	5900
0	1	5900
0	0	5901
0	1	5901
0	0	5902
0	1	5902
0	0	5903
0	1	5903
0	0	5904
0	1	5904
0	0	5905
0	1	5905
0	0	5906
0	1	5906
0	0	5907
0	1	5907
0	0	5908
0	1	5908
0	0	5909
0	1	5909
0	0	5910
0	1	5910
0	0	5911
0	1	5911
0	0	5912
0	1	5912
0	0	5913
0	1	5913
0	0	5914
0	1	5914
0	0	5915
0	1	5915
1	0	5916
0	1	5916
0	0	5917
0	1	5917
0	0	5918
0	1	5918

0	0	5919
0	1	5919
0	0	5920
0	1	5920
0	0	5921
0	1	5921
0	0	5922
0	1	5922
0	0	5923
0	1	5923
0	0	5924
0	1	5924
0	0	5925
0	1	5925
0	0	5926
0	1	5926
0	0	5927
0	1	5927
0	0	5928
0	1	5928
0	0	5929
0	1	5929
0	0	5930
0	1	5930
0	0	5931
0	1	5931
0	0	5932
0	1	5932
0	0	5933
1	1	5933
0	0	5934
0	1	5934
0	0	5935
0	1	5935
0	0	5936
0	1	5936
0	0	5937
0	1	5937
0	0	5938
0	1	5938
0	0	5939
0	1	5939
0	0	5940
0	1	5940
0	0	5941
0	1	5941
0	0	5942
0	1	5942
0	0	5943
0	1	5943
0	0	5944
0	1	5944
0	0	5945
0	1	5945
0	0	5946
0	1	5946
0	0	5947
0	1	5947
0	0	5948
0	1	5948
0	0	5949
0	1	5949
0	0	5950
0	1	5950
0	0	5951
0	1	5951
0	0	5952
0	1	5952
0	0	5953
0	1	5953
0	0	5954
0	1	5954
0	0	5955
0	1	5955
0	0	5956
0	1	5956
0	0	5957

0	1	5957
0	0	5958
0	1	5958
0	0	5959
0	1	5959
0	0	5960
0	1	5960
0	0	5961
0	1	5961
0	0	5962
0	1	5962
0	0	5963
0	1	5963
0	0	5964
0	1	5964
0	0	5965
0	1	5965
0	0	5966
1	1	5966
0	0	5967
0	1	5967
0	0	5968
0	1	5968
0	0	5969
0	1	5969
0	0	5970
0	1	5970
0	0	5971
0	1	5971
0	0	5972
0	1	5972
0	0	5973
0	1	5973
0	0	5974
0	1	5974
0	0	5975
0	1	5975
0	0	5976
0	1	5976
0	0	5977
0	1	5977
0	0	5978
0	1	5978
0	0	5979
0	1	5979
0	0	5980
0	1	5980
0	0	5981
0	1	5981
0	0	5982
0	1	5982
0	0	5983
0	1	5983
0	0	5984
0	1	5984
0	0	5985
0	1	5985
0	0	5986
0	1	5986
0	0	5987
0	1	5987
0	0	5988
0	1	5988
0	0	5989
0	1	5989
0	0	5990
0	1	5990
0	0	5991
0	1	5991
0	0	5992
0	1	5992
0	0	5993
0	1	5993
0	0	5994
0	1	5994
0	0	5995
0	1	5995

0	0	5996
0	1	5996
0	0	5997
0	1	5997
0	0	5998
0	1	5998
0	0	5999
0	1	5999
1	0	6000
0	1	6000
